# DNA Repair and Immune Response Pathways Are Deregulated in Melanocyte-Keratinocyte Co-cultures Derived From the Healthy Skin of Familial Melanoma Patients

**DOI:** 10.3389/fmed.2021.692341

**Published:** 2021-10-01

**Authors:** Miriam Potrony, Tariq Sami Haddad, Gemma Tell-Martí, Pol Gimenez-Xavier, Carlos Leon, Marta Pevida, Judit Mateu, Celia Badenas, Cristina Carrera, Josep Malvehy, Paula Aguilera, Sara Llames, Maria José Escámez, Joan A. Puig-Butillé, Marcela del Río, Susana Puig

**Affiliations:** ^1^Biochemistry and Molecular Genetics Department, Melanoma Unit, Hospital Clínic de Barcelona, Institut d'Investigacions Biomédiques August Pi i Sunyer, Universitat de Barcelona, Barcelona, Spain; ^2^Centro de Investigación Biomédica en Red de Enfermedades Raras, Instituto de Salud Carlos III, Madrid, Spain; ^3^Dermatology Department, Melanoma Unit, Hospital Clínic de Barcelona, Institut d'Investigacions Biomédiques August Pi i Sunyer, Universitat de Barcelona, Barcelona, Spain; ^4^Departamento de Bioingeniería, Universidad Carlos III de Madrid, Leganés, Spain; ^5^Cátedra de Medicina Regenerativa y Bioingeniería de Tejidos, Instituto de Investigación Sanitaria de la Fundación Jiménez Díaz, Madrid, Spain; ^6^Tissue Engineering Unit, Centro Comunitario de Sangre y Tejidos de Asturias, Oviedo, Spain; ^7^Instituto Universitario Fdez-Vega, Fundación de Investigación Oftalmológica, Universidad de Oviedo, Oviedo, Spain; ^8^Centro de Investigaciones Energéticas Mediambientales y Tecnonlógicas, Madrid, Spain; ^9^Molecular Biology Core, Biomedical Diagnostic Center, Melanoma Unit, Hospital Clínic de Barcelona, Institut d'Investigacions Biomédiques August Pi i Sunyer, University of Barcelona, Barcelona, Spain

**Keywords:** familial melanoma, genetics, transcriptome, skin, DNA repair, immune response, cancer

## Abstract

Familial melanoma accounts for 10% of cases, being *CDKN2A* the main high-risk gene. However, the mechanisms underlying melanomagenesis in these cases remain poorly understood. Our aim was to analyze the transcriptome of melanocyte-keratinocyte co-cultures derived from healthy skin from familial melanoma patients vs. controls, to unveil pathways involved in melanoma development in at-risk individuals. Accordingly, primary melanocyte-keratinocyte co-cultures were established from the healthy skin biopsies of 16 unrelated familial melanoma patients (8 *CDKN2A* mutant, 8 *CDKN2A* wild-type) and 7 healthy controls. Whole transcriptome was captured using the SurePrint G3 Human Microarray. Transcriptome analyses included: differential gene expression, functional enrichment, and protein-protein interaction (PPI) networks. We identified a gene profile associated with familial melanoma independently of *CDKN2A* germline status. Functional enrichment analysis of this profile showed a downregulation of pathways related to DNA repair and immune response in familial melanoma (*P* < 0.05). In addition, the PPI network analysis revealed a network that consisted of double-stranded DNA repair genes (including *BRCA1, BRCA2, BRIP1*, and *FANCA*), immune response genes, and regulation of chromosome segregation. The hub gene was *BRCA1*. In conclusion, the constitutive deregulation of *BRCA1* pathway genes and the immune response in healthy skin could be a mechanism related to melanoma risk.

## Introduction

Familial cancers are developed on the background of germline mutations when additional genetic alterations occur in oncogenes or tumor suppressor genes and are accumulated in somatic cells (1). While some high-risk cancer susceptibility genes are well-known, the mechanism behind this susceptibility is still not well-established.

Melanoma is the skin cancer arising from melanocytes. Familial melanoma accounts for around 10% of cases. To date, *CDKN2A* is the main high-risk gene mutated in 20–40% of families, although other high-risk genes have been identified in a lower percentage of families (2, 3). In a previous study (4), we performed healthy skin biopsies from two pairs of siblings belonging to melanoma-prone families. In each pair, one sibling carried a germline mutation in *CDKN2A* and the other was wild-type. We established keratinocyte and melanocyte co-cultures from the skin biopsies and studied the expression profile. We observed that the expression profile from healthy keratinocyte-melanocyte co-culture of *CDKN2A*-mutated individuals was more similar to melanoma tumor expression than wild-type (4). Normal skin fibroblast from *CDKN2A* mutation carriers also presented altered expression signatures compared to controls (5). Previous studies have found similar results with other familial cancer syndromes: both normal epithelial cells from *BRCA1* or *BRCA2* carriers [Breast and Ovarian hereditary syndrome, OMIM (Online Mendelian Inheritance in Man) #604370 and #612555] (6) and normal breast epithelial cells and stromal cells from *TP53* mutation carriers (Li-Fraumeni syndrome, OMIM #151623) (7) showed changes in gene expression. This data suggests that healthy cells from patients with a high risk of developing a particular type of cancer have specific gene profiles that can contribute as a risk factor to develop the tumor.

The use of protein-protein interaction (PPI) networks on differential gene expression datasets allows to highlight molecular mechanisms involved in the development of specific diseases or conditions (8), including melanoma (9). Our aim was to use this approach to explore differential gene expression profiles in healthy skin keratinocyte-melanocyte co-cultures from familial melanoma patients, including both *CDKN2A* mutated and *CDKN2A* wild-type affected members, to identify novel pathways involved in melanoma susceptibility.

## Materials and Methods

### Patients and Samples

The study included 16 unrelated melanoma patients (7 males, 9 females) with a family history of melanoma, visited at the Melanoma Unit of Hospital Clínic of Barcelona. Genetic testing for *CDKN2A, CDK4*, and *MITF* was performed in all patients as previously reported (10, 11). Healthy skin biopsies from non-sun-exposed forearm areas were obtained from 8 familial melanoma cases harboring *CDKN2A* germline mutations and 8 patients belonging to *CDKN2A* wild-type pedigrees. None of the patients carried mutations in *CDK4* or the p.Glu318Lys variant in *MITF*. All patients gave written informed consent. The project was approved by the ethical committee of Hospital Clínic of Barcelona (Ref. 2013/8305). Additionally, we included healthy skin from 7 healthy controls (5 males, 2 females), obtained from multiorgan donors (*N* = 5) and foreskin remnants after surgical treatment of phimosis (*N* = 2). Clinical information is detailed in Supplementary Table 1.

### Primary Melanocyte and Keratinocyte Co-culture Establishment

In order to harmonize biopsy conditions and focus on cell-expression differences based on the germline background, skin biopsies were processed to obtain primary melanocyte and keratinocyte co-cultures from all patients. To do so, we adapted the protocol used in our previous work (4). Skin biopsies from non-lesional and non-sun-exposed areas were processed by mechanical fragmentation and enzymatic digestion, with 0.25% trypsin – EDTA 0.02% (Sigma, 37°C, 3 cycles of 30 min), centrifuged (1,400 rpm for 10 min) and seeding of the sediment (2.5 × 104 cells/cm^2^) on a layer of 3T3 mouse fibroblasts lethally irradiated with Gamma-irradiation (60 Gy) seeded 24 h before (1 × 105 cells/cm^2^). Melanocytes and Keratinocytes were amplified once attached. The cultures were maintained at 37°C, saturated humidity atmosphere and 5% CO_2_. The cells were cryopreserved in liquid nitrogen carried out in DMEM with 15% SBF and 10% Glicerol (Sigma-Aldrich, Merck, Saint Louis, Missouri, US).

### Molecular Analyses

Total RNA isolation from primary cultures at early passages, was performed using the Trizol extraction method (Invitrogen Life Technologies, Carlsbad, USA). Total isolated RNA was purified using the RNeasy kit (Qiagen, Hilden, Germany). RNA concentration was determined using a NanoDrop Spectrophotometer (Thermo Fisher Scientific, Waltham, USA) and integrity of the RNA was verified using a Bioanalyzer 2100 (Agilent Technologies, Santa Clara, USA).

Analysis of global expression was performed using the SurePrint G3 Human gene expression 8 × 60 k v2 Microarray kit (G4851B) (Agilent, Santa Clara, CA, US). The microarray contains over 60,000 probes targeting more than 35,000 unique human mRNA coding genes and intergenic lncRNA. The Low Input Quickamp Labeling kit (Agilent, Santa Clara, CA, US) was used to label 50 ng of RNA. To standardize the results, 10 commercial control probes were added to all samples (RNA Spike-in kit, one color) (Agilent, Santa Clara, CA, US). The arrays were scanned using the DNA Microarray Scanner G2565CA (Agilent, Santa Clara, CA, US). Feature Extraction Software (Agilent, Santa Clara, CA, US) was used both to perform the quality control process and to extract the information. Cases (*CDKN2A* mutated and wild-type) and controls were distributed randomly in the arrays to avoid batch effect. Data has been uploaded to GEO database (Gene Expression Omnibus https://www.ncbi.nlm.nih.gov/geo/) with accession number GSE160902.

Among the differentially expressed genes, considering statistical significance, presence in multiple comparisons and biological functions, we selected 11 genes for quantitative PCR (qPCR) validation. Reverse transcription PCR (RT-PCR) was performed using the High-Capacity cDNA Reverse Transcription Kit (Thermofisher, Waltham, MA, US). We used commercial qPCR assays (IDT, Coralville, IA, US) and the TaqMan™ Universal PCR Master Mix (Thermofisher, Waltham, MA, US): *BRCA1* (Hs.PT.56a.27724517.g), *CYP2J2* (Hs.PT.58.28125130), *EIF2AK2* (Hs.PT.58.2657801), *HEPH* (Hs.PT.58.2449607), *IRF9* (Hs.PT.58.3264634), *NMI* (Hs.PT.58.38727825), *OAS1* (Hs.PT.58.2338899), *PARP9* (Hs.PT.58.14456843), *RAD51D* (Hs.PT.58.18812973), *SEMA3D* (Hs.PT.58.3096304), *STAT1* (Hs.PT.58.15049687), and housekeeping genes *GUSB* (Hs.PT.58v.27737538), *PPIA* (Hs.PT.58v.38887593.g), and *TBP* (Hs.PT.58v.39858774). Amplification was performed in a 7900HT Fast Real-Time PCR system (Thermofisher, Waltham, MA, US) with 389 well plates. We performed three technical replications for each gene and sample and used a reference sample for normalization.

### Data Analysis

Whole transcriptome expression data analysis was carried out using the free tool Babelomics 5.0 (12). The following comparisons were performed: samples from all melanoma patients vs. controls, samples from *CDKN2A* carriers vs. controls, samples from *CDKN2A* wild-type patients vs. controls, and *CDKN2A* carriers vs. *CDKN2A* wild-type. Additionally, analyses by sex were performed (Supplementary Material). After normalization and duplicate elimination, principal components analysis (PCA) and euclidean clusterization were performed using R for each comparison. Differential gene expression analysis was conducted using the limma statistical package from Bioconductor. *P*-values were adjusted for multiple testing according to the Benjamini and Hochberg procedure for controlling the False Discovery Rate (FDR = 5%). Heatmaps from the significant deregulated genes were generated using R packages gplots and heatmap.plus.

Gene set analysis was carried out using FatiScan (13) in Babelomics 5.0 to identify significant Gene Ontologies (GOs) overrepresented in the set of significant genes. The significant Kyoto Encyclopedia of Genes and Genomes pathways (KEGG) were assessed from the KEGG website (https://www.genome.jp/kegg/) (14).

Protein-protein interaction (PPI) networks were created based on the genes resulting from the differential expression analysis (9). The identification of statistically significant PPI networks was conducted through SNOW implemented within Babelomics 5.0. In order to evaluate the cooperative behavior of a list of genes as a functional module, protein-protein interaction data from IntAct Molecular Interaction Database, Biomolecular Interaction Network Database, Database of Interacting Proteins, Human Protein Reference Database, and Molecular INTeraction database. SNOW calculates the minimal connected network (MCN) of the gene list and then compares the topology of this MCN against 10,000 random MCNs with the same size range to obtain a *p*-value. For network significance, we focused on connectivity, which has been shown to be a robust indicator of having a functional module (15). Network isualization and evaluation was done using Cytoscape (16).

For qPCR data analyses the 2^ΔΔCt^ method was used. Case and control 2^ΔΔCt^ values were compared using the non-parametric Mann-Whitney U test. Results were considered statistically significant when *p*-values were <0.05. Statistical analyses were performed using IBM SPSS software (version 25).

## Results

**Figure 1:**
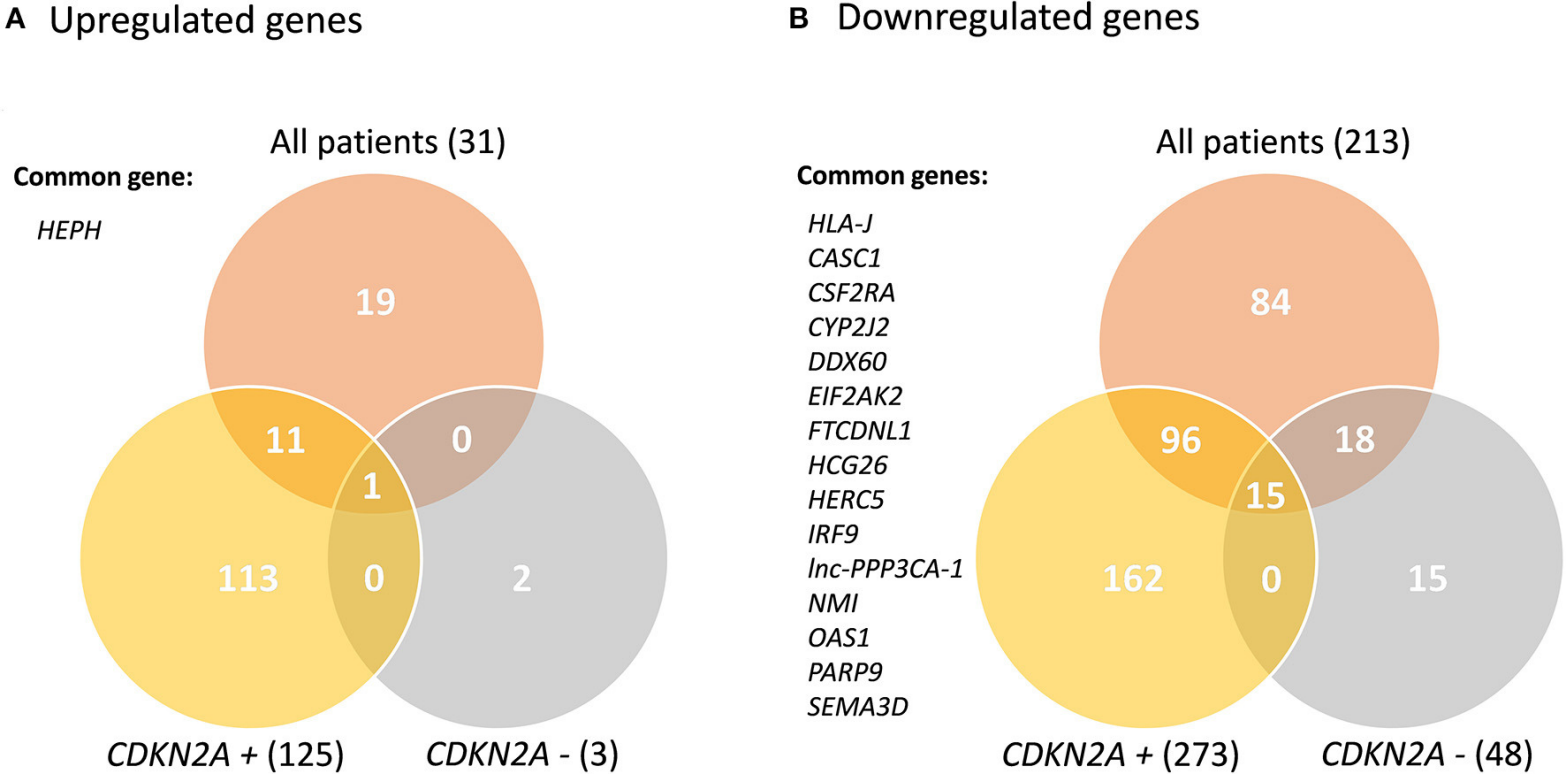
Venn Diagram of the significant deregulated genes in all comparisons. *CDKN2A* +: mutated; *CDKN2A* –: wild-type. Common genes between the three comparisons are listed.

Transcriptome analyses of primary keratinocyte-melanocyte co-cultures derived from healthy skin biopsies from all familial melanoma patients vs. controls identified differential gene profiles (Supplementary Figure 1). In this global comparison, we identified 244 significantly deregulated transcripts in melanoma cases (31 up-regulated, 213 downregulated). From these, 210 were unique differentially expressed genes (DEGs) (19 upregulated, 191 downregulated). In the *CDKN2A*-based analyses, we identified 316 unique DEGs in *CDKN2A* mutation carriers vs. controls (73 upregulated, 243 downregulated) and 43 unique DEGs in *CDKN2A* wild-type patients vs. controls (2 upregulated, 41 downregulated) (Supplementary Tables 2–4). No significant DEGs were identified comparing melanoma patients with or without *CDKN2A* mutation. One gene was upregulated (*HEPH*) and 15 genes were downregulated in the three significant comparisons (*HLA-J, CASC1, CSF2RA, CYP2J2, DDX60, EIF2AK2, FTCDNL1, HCG26, HERC5, IRF9, lnc-PPP3CA-1, NMI, OAS1, PARP9*, and *SEMA3D*) (Figure 1). These results were not sex-related (Supplementary Material).

We selected *BRCA1, CYP2J2, EIF2AK2, HEPH, IRF9, NMI, OAS1, PARP9, RAD51D, SEMA3D*, and *STAT1* genes for validation of the results seen in the array analyses by qPCR. We compared 2^Δ^ΔCt values in cases and controls using a non-parametric statistical test. We found a significant upregulation of *HEPH* and significant downregulation of the rest of genes (*BRCA1, CYP2J2, EIF2AK2, IRF9, NMI, OAS1, PARP9, SEMA3D*, and *STAT1*) except for *RAD51D* where the difference that did not reach statistical significance (*p* = 0.089; Supplementary Figure 2).

Functional enrichment analyses of significant DEGs in the global comparison revealed an overrepresentation of downregulated genes in Fanconi anemia, homologous recombination, mismatch repair and DNA replication KEGG pathways, as well as several immune response KEGG pathways (Supplementary Table 5). There was a high correlation between significant KEGG pathways and significant GO terms (Supplementary Table 6).

**Figure 2:**
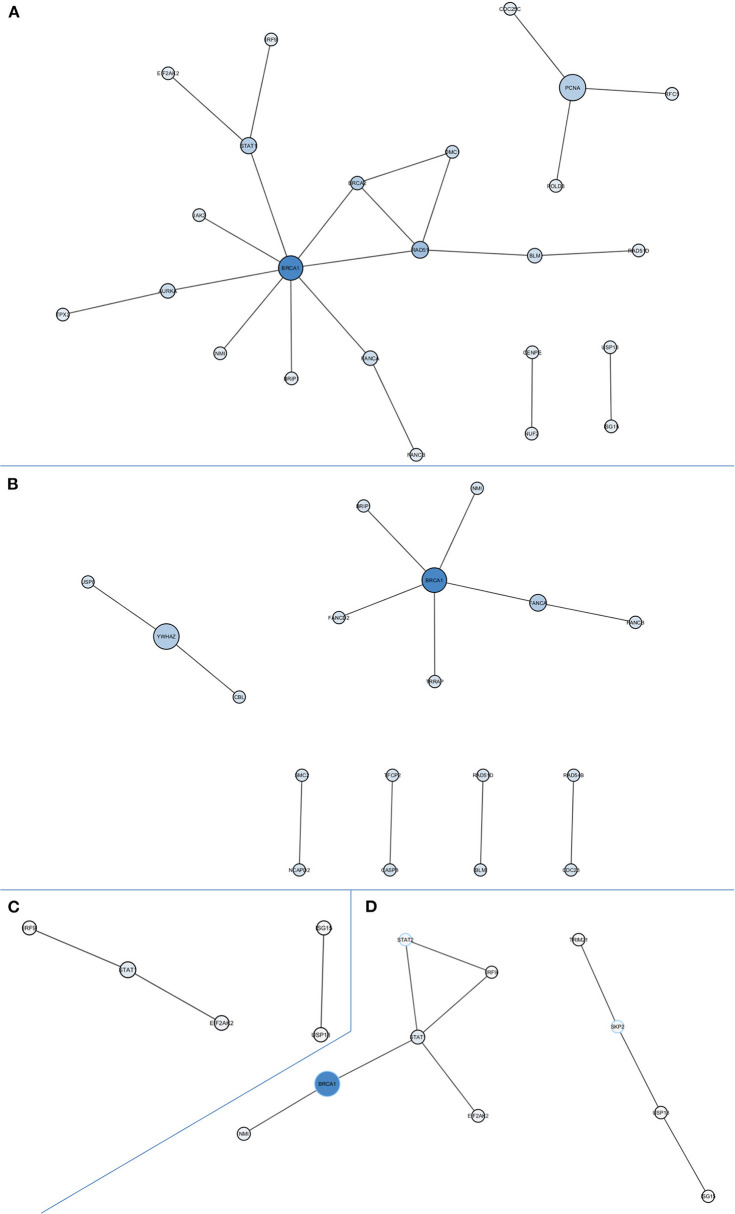
Protein-protein interaction networks of downregulated differentially expressed genes in all comparisons. **(A)** All melanoma patients vs. controls without intermediates, network *P* = 0.0005; **(B)** *CDKN2A* mutant patients vs. controls without intermediates, network *P* = 0.0001; **(C)** *CDKN2A* wild-type patients vs. controls without intermediates, network *P* = 0.0375; **(D)** *CDKN2A* wild-type patients vs. controls with intermediates, network *P* = 0.0116. Size of each node depends on the number of connections and betweenness centrality in the network.

PPI network-based analysis of downregulated DEGs in the global comparison identified a significant network composed by 24 nodes with at least one degree of connection without using intermediates (*P* = 0.0005; Figure 2A). The hub gene, defined as the gene with the highest degree of connection and the highest betweenness centrality, was *BRCA1*. Globally, the genes in this network are involved in homologous recombination and Fanconi anemia pathways (*BLM, BRCA1, BRCA2, BRCC3, BRIP1, DMC1, FANCA, RAD51, RAD51D*), DNA repair and replication (*PCNA, RFC5, POLD3*), microtubule formation and the regulation of chromosome segregation (*AURKA, TPX2, CENPE, NUF2*), and immune response (*EIF2AK2, IRF9, JAK2, NMI, STAT1, USP18, ISG15, CDC25C*). We also assessed the PPI networks of significant DEGs in the *CDKN2A*-based analyses. We identified a significant network composed of 18 nodes with at least one degree of connection without intermediates (*P* = 0.0001) in DEGs downregulated in *CDKN2A* mutant patients (Figure 2B). In this case, *BRCA1* was also the hub gene. Finally, in *CDKN2A* wild-type patients, without using intermediates, a PPI network with immune response genes was identified (Figure 2C). And when using intermediates, *BRCA1* was also the gene with highest betweenness centrality of the network identified (*P* = 0.0116; Figure 2D).

One limitation of this study might be the differences in age at biopsy between patients and controls [48.6 years old (range 25–68) SD = 9.9 and 22.1 years old (range 5–54) SD = 18.1, respectively]. To evaluate this, we compared our DEGs with those detected in previous studies assessing age-related gene expression profiles in skin (17, 18). Overall, 80–89% of our DEGs were not associated with skin aging (Supplementary Table 7). Expression levels of *BRCA1*, our hub gene in the network analyses, were neither found to be associated with skin aging. These results support that our findings are specific to melanoma rather than as a consequence of the age difference between patients and controls.

Additionally, to discard possible biases associated with variations in the melanocyte/keratinocyte ratio in the culture, we compared the expression of keratinocyte-specific genes (*KRT6A, KRT5*, and *KRT14*) and melanocyte specific genes (*DCT, MC1R*, and *MITF*) in the different study groups. We did not observe differences in normalized log10 gene expression levels between controls, *CDKN2A* mutant or *CDKN2A* wild-type familial melanoma patients (Supplementary Figure 3). Therefore, the melanocyte/keratinocyte proportion is constant in the samples and does not influence the results obtained.

Finally, we checked whether our DEGs were previously identified in studies that compared normal and tumor tissue, both in melanoma and breast cancer. Twelve of our genes were also deregulated in breast tumor tissue compared with normal adjacent tissue (*CCDC50, CPNE8, FAM122B, GNL1, HN1, ICA1L, IFITM4P, JAK2, KIAA1841, STXBP5, TTC13, ZNF333*) (19). From studies comparing melanoma and nevi, only one common gene was found (*HN1*) in Scatolini and collaborators publication (20), but no common genes were identified between our study and that by Haqq et al. (21). However, when focusing not on gene expression, but on literature matches or genetic data available relating our DEGs to melanoma or breast cancer, 60 and 39% DEGs have been previously associated with breast cancer and melanoma, respectively [data obtained from Open Target Platform https://platform.opentargets.org/ (22)] (Supplementary Table 8).

## Discussion

In our study we evaluated the differential gene expression of keratinocyte-melanocyte co-cultures obtained from the healthy skin of familial melanoma patients, compared with co-cultures from healthy controls. Considering *CDKN2A* is the major susceptibility gene in familial melanoma but is only mutated in around 20–40% of families worldwide, half of the patients included were *CDKN2A* wild-type. In our study, we have not detected significant differences between familial melanoma patients regarding *CDKN2A* status. Comparable results were detected in *CDKN2A*-based analyses and global comparison. Thus, the differences in genetic susceptibility in individuals at high melanoma risk seem to be due to the deregulation of similar pathways.

Both functional enrichment analyses and PPI network analyses of downregulated genes in primary keratinocyte-melanocyte co-cultures from familial melanoma pointed to homologous recombination and Fanconi anemia pathways as potential players in melanoma susceptibility. These pathways participate in a vital DNA repair process by repairing double-stranded DNA breaks. Such breaks can occur during DNA replication, when the genome is particularly susceptible to DNA damage, or from exposure to ionizing radiation and other genotoxic substances. When the homologous recombination pathway is not working properly, DNA breaks can result in chromosome instability enhancing genomic instability and tumorigenesis (23).

*BRCA1* was the hub gene in all network analyses. *BRCA1* and *BRCA2* (also present in the global downregulated network) are tumor suppressor genes belonging to the homologous recombination and Fanconi anemia pathways, involved in preserving chromosomal stability, regulating cell response to double-stranded DNA breaks and participating in DNA repair. *BRCA1* and *BRCA2* are the main susceptibility genes in breast and ovarian familial cancer syndrome. There exists an epidemiological relationship between susceptibility to both breast cancer and melanoma, suggesting a shared molecular basis at some level. Although it remains a controversial issue, several studies have shown an increased prevalence of melanoma also among *BRCA2* mutation carriers (24). Families with *CDKN2A* germline mutations show an increased prevalence of other cancers beyond melanoma, including breast cancer (10). Consequently, high-risk genes predisposing the development of one of these tumor types may also contribute to the development of another under circumstances not yet fully understood. Additionally, a family history of breast cancer is associated with having a high nevi count in their children (the main phenotypic risk factor for melanoma) (25). Based on epidemiological data and the known effect of germline variants in genes belonging to these DNA repair pathways on cancer susceptibility, the downregulation of these pathways in healthy skin could be a plausible melanoma susceptibility factor, independently of the molecular origin of this deregulation.

UV radiation is the main environmental risk factor in melanoma. Accordingly, one of the main genetic signatures in melanoma tumors shows a higher prevalence of C > T mutations consistent with the formation of pyrimidine dimers (26), which are the lesions repaired by the nucleotide excision repair mechanism. However, the loss of double strand break repair mechanisms can lead to an increased frequency of chromosomal rearrangements resulting in a loss or gain of genetic material (27), also observed in melanoma (28, 29). Thus, the deregulation of this pathway could play an important role in melanoma development.

Another important player in melanoma susceptibility based on functional enrichment analyses and PPI network analyses could well be the deregulation of the immune response. Melanoma is a highly immunogenic tumor and immunotherapy is today used to treat advanced melanoma patients achieving durable responses (30). Paradoxically, the immune system participates in tumor development, beyond its role in prognosis. UV exposure induces immune suppression, which has been shown to play a critical role in skin cancer induction (31). Immunosuppressed individuals, such as organ transplant recipients, have an increased risk of developing melanoma (32). Moreover, low-penetrance polymorphisms modulating immune responses have been associated with melanoma susceptibility (33). Based on this evidence, it is plausible that basal deregulation of immune responses in the skin can act as a risk factor for melanoma development.

Our results reinforce the idea that the study of expression profiles in the healthy tissues of patients at risk of developing cancer can lead to the identification of new pathways involved in cancer susceptibility. If these results are confirmed in skin biopsies from larger prospective cohorts, in the future, when no germline mutations are detected in known susceptibility genes, gene expression profiling of normal tissue may give a clue to who is at risk of developing melanoma in the families.

In conclusion, the gene expression pattern in the healthy skin of individuals at high risk of developing melanoma could be in itself a risk factor for melanoma development. Our study suggests that skin downregulation of homologous recombination and Fanconi anemia pathways, together with the deregulation of the immune response, may well be melanoma susceptibility factors.

## Data Availability Statement

The datasets presented in this study can be found in online repositories. The names of the repository/repositories and accession number(s) can be found below: https://www.ncbi.nlm.nih.gov/geo/, GSE160902.

## Ethics Statement

The studies involving human participants were reviewed and approved by Ethical Committee of Hospital Clínic of Barcelona. Written informed consent to participate in this study was provided by the patient or the participants' legal guardian/next of kin.

## Author Contributions

SP, JP-B, ME, and MR contributed to the conception and design of the work. MPo, TH, and PG-X contributed to data analysis and interpretation. GT-M, CL, MPe, JMal, CB, CC, JMat, PA, and SL contributed to the acquisition of data. MPo performed the general drafting of the manuscript. All authors reviewed the manuscript and gave their final approval of the version submitted.

## Funding

The main funding of this project came from the intramural project *Papel del estrés oxidativo en el desarrollo de Melanoma Familiar y otras ER comunes con predisposición al desarrollo de neoplasias cutáneas* financed by Centro de Investigación Biomédica en Red de Enfermedades Raras (CIBERER), of the Instituto de Salud Carlos III, Spain, co-financed by European Development Regional Fund A way to achieve Europe ERDF. The research at the Melanoma Unit in Barcelona is partially funded by Spanish Fondo de Investigaciones Sanitarias Grants PI15/00716 and PI15/00956, of the Instituto de Salud Carlos III, Spain, co-financed by European Development Regional Fund A way to achieve Europe ERDF; AGAUR 2017_SGR_1134 of the Catalan Government, Spain; European Commission under the 6th Framework Programme, Contract No. LSHC-CT-2006-018702 (GenoMEL) and by the European Commission under the 7th Framework Programme, Diagnoptics; The National Cancer Institute (NCI) of the US National Institute of Health (NIH) (CA83115); a grant from Fundació La Marató de TV3 201331-30, Catalonia, Spain; a grant from Fundación Científica de la Asociación Española Contra el Cáncer GCB15152978SOEN, Spain, and CERCA Programme/Generalitat de Catalunya. Part of the work was carried out at the Esther Koplowitz Center, Barcelona. The UC3M-CIEMAT-CIBERER-IISFJD research is mainly supported by grants from the Spanish Ministry of Economy and Competitiveness (SAF2017-86810-R) and from the Community of Madrid (AvanCell-CM S2017/BMD-3692) which are co-funded with European Regional Development Funds (ERDF). TH was currently recipient of a PhD Fellowship at Radboud University Medical Center in the Netherlands funded by the Dutch Cancer Society (KWF) (10602).

## Conflict of Interest

The authors declare that the research was conducted in the absence of any commercial or financial relationships that could be construed as a potential conflict of interest.

## Publisher's Note

All claims expressed in this article are solely those of the authors and do not necessarily represent those of their affiliated organizations, or those of the publisher, the editors and the reviewers. Any product that may be evaluated in this article, or claim that may be made by its manufacturer, is not guaranteed or endorsed by the publisher.
