# Supplementary material for: DNA Repair and Immune Response Pathways Are Deregulated in Melanocyte-Keratinocyte Co-cultures Derived From the Healthy Skin of Familial Melanoma Patients
Source: Front Med (Lausanne). 2021 Oct 1;8:692341. doi: 10.3389/fmed.2021.692341 (PMC8517393; doi:10.3389/fmed.2021.692341)
Supplement: Supplementary file 1 [file Data_Sheet_1.DOCX]

Supplementary Material

INDEX Page

# 1. Supplementary Figures and Tables 2

## 1.1 Supplementary Tables 2

Table S1. Clinical characteristics of individuals included in the study 2

Table S2. Differentially expressed genes in familial melanoma patients vs controls 4

Table S3. Differentially expressed genes in *CDKN2A* mutated familial melanoma patients vs

controls 11

Table S4. Differentially expressed genes in *CDKN2A* wild-type familial melanoma patients vs

controls 22

Table S5. Significant KEEGs overrepresented in downregulated genes in familial melanoma patients

vs controls 24

Table S6. Top 20 (of 338) significant GOs overrepresented in downregulated genes in familial

melanoma patients vs controls 25

Table S7. DEGs identified in our study and previous association with aging 26

Table S8. Open Targets platform association score for each significant DEG in breast cancer and melanoma phenotype 40

**1.2 Supplementary Figures** 56

Supplementary Figure 1. Principal components analysis and heatmap of the significant differentially expressed genes according to comparisons. 56

Supplementary Figure 2. qPCR results boxplot. 58

Supplementary Figure 3. Log10 gene expression of keratinocyte and melanocyte specific genes among study groups. 59

**2. Supplementary data** 60

**2.1 Analyses by sex** 60

# Supplementary Figures and Tables

## Supplementary Tables

**Table S1. Clinical characteristics of individuals included in the study**

| **ID** | **Sex** | **Age at biopsy** | **Melanoma** | **Age at diagnosis** | **Number of primary melanomas** | **Number of cases in the family** | **CDKN2A status** | **Control type** |
| --- | --- | --- | --- | --- | --- | --- | --- | --- |
| M1 | M | 35 | Yes | 21 | 1 | 5 | WT | - |
| M2 | M | 43 | Yes | 32 | 2 | 2 | WT | - |
| M3 | F | 67 | Yes | 50 | 2 | 4 | Mutated | - |
| M4 | M | 49 | Yes | 27 | 3 | 2 | Mutated | - |
| M5 | M | 64 | Yes | 52 | 3 | 2 | WT | - |
| M6 | F | 41 | Yes | 33 | 1 | 3 | Mutated | - |
| M7 | F | 41 | Yes | 24 | 1 | 3 | Mutated | - |
| M8 | F | 44 | Yes | 31 | 3 | 2 | WT | - |
| M9 | F | 40 | Yes | 37 | 2 | 2 | WT | - |
| M10 | M | 54 | Yes | 36 | 1 | 9 | Mutated | - |
| M11 | M | 47 | Yes | 39 | 2 | 2 | WT | - |
| M12 | F | 47 | Yes | 35 | 2 | 2 | Mutated | - |
| M13 | F | 42 | Yes | 36 | 3 | 4 | Mutated | - |
| M14 | M | 49 | Yes | 41 | 2 | 3 | Mutated | - |
| M15 | F | 47 | Yes | 45 | 7 | 3 | WT | - |
| M16 | F | 68 | Yes | 61 | 2 | 3 | WT | - |
| C1 | M | 5 | No | - | - | - | - | foreskin remnant after surgical treatment of phimosis |
| C2 | M | 5 | No | - | - | - | - | foreskin remnant after surgical treatment of phimosis |
| C3 | F | 19 | No | - | - | - | - | Multiorganic donnor, car accident |
| C4 | M | 18 | No | - | - | - | - | Multiorgan donor, cranioencefalic traumatism |
| C5 | M | 39 | No | - | - | - | - | Multiorgan donor, toracoabdominal traumatism in a work-accident |
| C6 | M | 15 | No | - | - | - | - | Multiorgan donor, cranioencefalic traumatism |
| C7 | F | 54 | No | - | - | - | - | Multiorgan donor, brain hemorrhage |

M: male, F: female; WT: wild-type

**Table S2. Differentially expressed genes in familial melanoma patients vs controls**

| **Gene ID** | **statistic** | **p-value** | **adj. p-value** |
| --- | --- | --- | --- |
| HEPH | 5.9115124 | 4.93E-06 | 0.01137252 |
| KLRG2 | 5.66971926 | 8.86E-06 | 0.0139349 |
| HSPG2 | 5.37345043 | 1.83E-05 | 0.01710565 |
| C14orf180 | 5.33719579 | 2.00E-05 | 0.01792336 |
| CTSB | 5.32434376 | 2.07E-05 | 0.01812242 |
| GPC6 | 5.22232553 | 2.66E-05 | 0.01985321 |
| SELM | 5.2171819 | 2.69E-05 | 0.01985321 |
| LMX1B | 5.16074304 | 3.09E-05 | 0.01985321 |
| MECOM | 4.93043722 | 5.48E-05 | 0.02621452 |
| RNVU1-18 | 4.91663989 | 5.67E-05 | 0.02645225 |
| LINGO2 | 4.76837205 | 8.21E-05 | 0.03067675 |
| WDR86 | 4.67974551 | 1.02E-04 | 0.0327952 |
| TNFRSF10D | 4.66276023 | 1.07E-04 | 0.03279842 |
| CCDC88C | 4.5801366 | 1.31E-04 | 0.03653757 |
| LMF1 | 4.53834404 | 1.46E-04 | 0.03815107 |
| IRX1 | 4.48699948 | 1.66E-04 | 0.04038244 |
| LAMP2 | 4.38594598 | 2.13E-04 | 0.04448749 |
| VPREB3 | 4.35638739 | 2.30E-04 | 0.04550124 |
| TMEM145 | 4.34390892 | 2.37E-04 | 0.04617144 |
| MELK | -4.2795976 | 2.78E-04 | 0.0490881 |
| SLFN12 | -4.2797757 | 2.78E-04 | 0.0490881 |
| DDIAS | -4.2800761 | 2.78E-04 | 0.0490881 |
| RDM1 | -4.2858739 | 2.74E-04 | 0.0490881 |
| MIS18A | -4.2861999 | 2.74E-04 | 0.0490881 |
| ACLY | -4.2922601 | 2.70E-04 | 0.04890179 |
| CSE1L | -4.2923754 | 2.70E-04 | 0.04890179 |
| ASPG | -4.2929669 | 2.69E-04 | 0.04890179 |
| C1orf112 | -4.2931362 | 2.69E-04 | 0.04890179 |
| FANCB | -4.3078512 | 2.59E-04 | 0.04804722 |
| GINS2 | -4.3106467 | 2.57E-04 | 0.04791934 |
| MOCS2 | -4.3144832 | 2.55E-04 | 0.04766855 |
| PHF11 | -4.3148335 | 2.55E-04 | 0.04766855 |
| ACTR3 | -4.3199218 | 2.52E-04 | 0.04743765 |
| DMC1 | -4.3238034 | 2.49E-04 | 0.04718673 |
| IFIH1 | -4.3242915 | 2.49E-04 | 0.04718673 |
| FANCI | -4.3307903 | 2.45E-04 | 0.04678224 |
| C2CD5 | -4.341421 | 2.38E-04 | 0.04617144 |
| SFR1 | -4.3416802 | 2.38E-04 | 0.04617144 |
| ZFP69 | -4.3446111 | 2.37E-04 | 0.04617144 |
| ADAMTSL3 | -4.357461 | 2.29E-04 | 0.04550124 |
| C8orf48 | -4.3603864 | 2.27E-04 | 0.04546783 |
| XRCC3 | -4.3617659 | 2.27E-04 | 0.04546783 |
| NCAPD2 | -4.3634321 | 2.26E-04 | 0.04546783 |
| KIF20B | -4.3687211 | 2.23E-04 | 0.04516066 |
| PSMB8 | -4.369927 | 2.22E-04 | 0.04516066 |
| COMMD8 | -4.3714095 | 2.21E-04 | 0.04516066 |
| IGF2BP3 | -4.3729849 | 2.20E-04 | 0.04516066 |
| IFIT1 | -4.3754539 | 2.19E-04 | 0.04516066 |
| PLAC1 | -4.3854059 | 2.14E-04 | 0.04448749 |
| FMO5 | -4.386297 | 2.13E-04 | 0.04448749 |
| ORC6 | -4.3883709 | 2.12E-04 | 0.04448749 |
| TPX2 | -4.4001261 | 2.06E-04 | 0.04425651 |
| FOLH1B | -4.4046131 | 2.04E-04 | 0.04398286 |
| TCEAL4 | -4.4076926 | 2.02E-04 | 0.04386605 |
| DHTKD1 | -4.4099532 | 2.01E-04 | 0.0438403 |
| CENPI | -4.421492 | 1.95E-04 | 0.04281124 |
| TBC1D8B | -4.4228722 | 1.95E-04 | 0.04281124 |
| BEND6 | -4.4258448 | 1.93E-04 | 0.04281124 |
| POLD3 | -4.4283951 | 1.92E-04 | 0.04281124 |
| CASC5 | -4.4337344 | 1.89E-04 | 0.04260797 |
| ASPM | -4.4480145 | 1.83E-04 | 0.04177 |
| NCAPD3 | -4.4481942 | 1.83E-04 | 0.04177 |
| SCEL | -4.459915 | 1.77E-04 | 0.04098178 |
| TMEM253 | -4.46272 | 1.76E-04 | 0.04091542 |
| ERI2 | -4.466058 | 1.75E-04 | 0.04079598 |
| HOXA7 | -4.4664638 | 1.74E-04 | 0.04079598 |
| EMP1 | -4.473153 | 1.72E-04 | 0.04051929 |
| LOC388282 | -4.4767108 | 1.70E-04 | 0.04038244 |
| ZWILCH | -4.4767596 | 1.70E-04 | 0.04038244 |
| DSCC1 | -4.4793997 | 1.69E-04 | 0.04038244 |
| RAD51 | -4.4848517 | 1.67E-04 | 0.04038244 |
| TRIM36 | -4.4878351 | 1.65E-04 | 0.04038244 |
| MCM8 | -4.489168 | 1.65E-04 | 0.04038244 |
| FAM129A | -4.4964964 | 1.62E-04 | 0.04021124 |
| ICA1L | -4.4991006 | 1.61E-04 | 0.04018261 |
| JAK2 | -4.5045597 | 1.59E-04 | 0.03986987 |
| AURKB | -4.5139731 | 1.55E-04 | 0.03917184 |
| TNNI3 | -4.5289803 | 1.49E-04 | 0.03840761 |
| FGFR1OP | -4.534065 | 1.47E-04 | 0.03815107 |
| ATAD5 | -4.5350025 | 1.47E-04 | 0.03815107 |
| ESCO2 | -4.5368126 | 1.46E-04 | 0.03815107 |
| LINC01133 | -4.5453726 | 1.43E-04 | 0.03815107 |
| ARPC2 | -4.5568744 | 1.39E-04 | 0.03762375 |
| UBE2L6 | -4.5680218 | 1.35E-04 | 0.03705629 |
| RTKN2 | -4.5704566 | 1.35E-04 | 0.03705629 |
| MCM6 | -4.5787943 | 1.32E-04 | 0.03653757 |
| KIF14 | -4.5848997 | 1.30E-04 | 0.0364548 |
| PARP12 | -4.586614 | 1.29E-04 | 0.0364548 |
| APC | -4.5932403 | 1.27E-04 | 0.03640849 |
| CDC25C | -4.5965274 | 1.26E-04 | 0.0363616 |
| CKAP2 | -4.6156528 | 1.20E-04 | 0.03489933 |
| IFI44 | -4.6199214 | 1.19E-04 | 0.03476406 |
| FERMT1 | -4.6218026 | 1.18E-04 | 0.03476406 |
| CENPA | -4.6271127 | 1.17E-04 | 0.03461612 |
| HMGN5 | -4.6296555 | 1.16E-04 | 0.03461612 |
| SEMA4D | -4.6398319 | 1.13E-04 | 0.03424213 |
| PLSCR1 | -4.6640493 | 1.06E-04 | 0.03279842 |
| ME1 | -4.6710465 | 1.05E-04 | 0.03279842 |
| XAF1 | -4.6744084 | 1.04E-04 | 0.0327952 |
| BORA | -4.6761078 | 1.03E-04 | 0.0327952 |
| SNX6 | -4.6817829 | 1.02E-04 | 0.0327952 |
| TWF1 | -4.6839371 | 1.01E-04 | 0.0327952 |
| SASS6 | -4.6896597 | 9.99E-05 | 0.0327952 |
| KLHL12 | -4.6959537 | 9.83E-05 | 0.03276413 |
| lnc-ITGA2-1 | -4.7033465 | 9.65E-05 | 0.03241633 |
| TMPRSS11D | -4.7039257 | 9.64E-05 | 0.03241633 |
| STAT1 | -4.7055766 | 9.60E-05 | 0.03241633 |
| IFI6 | -4.7065189 | 9.58E-05 | 0.03241633 |
| CLSPN | -4.7101609 | 9.49E-05 | 0.03241633 |
| GP6 | -4.7349493 | 8.92E-05 | 0.0311756 |
| FAM72D | -4.7419876 | 8.76E-05 | 0.03088396 |
| ODF3L1 | -4.7443123 | 8.71E-05 | 0.03088396 |
| NUF2 | -4.7473864 | 8.65E-05 | 0.03088396 |
| BRIP1 | -4.749515 | 8.60E-05 | 0.03088396 |
| EFCAB11 | -4.7565597 | 8.45E-05 | 0.03079087 |
| DSCAM | -4.7578054 | 8.42E-05 | 0.03079087 |
| IFI44L | -4.761982 | 8.34E-05 | 0.03079087 |
| ANP32E | -4.7694918 | 8.18E-05 | 0.03067675 |
| MRPS14 | -4.7821251 | 7.93E-05 | 0.03016699 |
| RFC5 | -4.7830677 | 7.91E-05 | 0.03016699 |
| AIM2 | -4.7873784 | 7.83E-05 | 0.03016699 |
| PCNA | -4.8027206 | 7.53E-05 | 0.02943844 |
| HLA-F | -4.8139037 | 7.33E-05 | 0.02889172 |
| AURKA | -4.8167507 | 7.27E-05 | 0.02889172 |
| PLIN4 | -4.8263393 | 7.10E-05 | 0.02853348 |
| CENPE | -4.8416189 | 6.84E-05 | 0.02799064 |
| HLA-G | -4.8444523 | 6.79E-05 | 0.02799064 |
| RNF180 | -4.865245 | 6.45E-05 | 0.02690314 |
| CMPK2 | -4.8709212 | 6.35E-05 | 0.02678552 |
| SP110 | -4.8740932 | 6.30E-05 | 0.02678552 |
| C6orf211 | -4.8778894 | 6.25E-05 | 0.02678552 |
| OAS2 | -4.8826754 | 6.17E-05 | 0.02678552 |
| EXO1 | -4.890194 | 6.06E-05 | 0.02657245 |
| LINC01510 | -4.8921011 | 6.03E-05 | 0.02657245 |
| ECT2 | -4.8923519 | 6.02E-05 | 0.02657245 |
| SHCBP1 | -4.8947081 | 5.99E-05 | 0.02657245 |
| HERC6 | -4.8981502 | 5.94E-05 | 0.02657245 |
| BRCA2 | -4.913053 | 5.72E-05 | 0.02645225 |
| TAP1 | -4.9198524 | 5.63E-05 | 0.02645225 |
| TICRR | -4.929858 | 5.49E-05 | 0.02621452 |
| FOLH1 | -4.9312539 | 5.47E-05 | 0.02621452 |
| BRCA1 | -4.936402 | 5.40E-05 | 0.02621452 |
| TRIM14 | -4.9370355 | 5.39E-05 | 0.02621452 |
| OAS2 | -4.9406273 | 5.34E-05 | 0.02621452 |
| LAMP3 | -4.953073 | 5.18E-05 | 0.02621452 |
| TUB | -4.9548932 | 5.16E-05 | 0.02621452 |
| IQCH | -4.9579442 | 5.12E-05 | 0.02621452 |
| USP18 | -4.9672004 | 5.00E-05 | 0.02621452 |
| GSAP | -4.9759997 | 4.89E-05 | 0.02621452 |
| CASP8 | -4.9773617 | 4.88E-05 | 0.02621452 |
| DNAAF3 | -5.0087752 | 4.51E-05 | 0.02518178 |
| SLC27A6 | -5.0121767 | 4.47E-05 | 0.02518178 |
| PTPN13 | -5.0149765 | 4.44E-05 | 0.02518178 |
| IL15RA | -5.0425031 | 4.15E-05 | 0.02409745 |
| FANCA | -5.0475715 | 4.10E-05 | 0.02409745 |
| MLLT4-AS1 | -5.0554182 | 4.02E-05 | 0.02398551 |
| PRTFDC1 | -5.0713626 | 3.86E-05 | 0.02337986 |
| APITD1 | -5.0862172 | 3.72E-05 | 0.02285603 |
| LAP3 | -5.1073256 | 3.53E-05 | 0.02200501 |
| TRAIP | -5.1176584 | 3.44E-05 | 0.02176407 |
| BLM | -5.1646607 | 3.06E-05 | 0.01985321 |
| PDCD1LG2 | -5.17176 | 3.01E-05 | 0.01985321 |
| HN1 | -5.1729984 | 3.00E-05 | 0.01985321 |
| CATSPER3 | -5.1815323 | 2.94E-05 | 0.01985321 |
| TTK | -5.1938043 | 2.85E-05 | 0.01985321 |
| HLA-J | -5.2002089 | 2.81E-05 | 0.01985321 |
| FAM111B | -5.2184181 | 2.68E-05 | 0.01985321 |
| DUSP19 | -5.2199608 | 2.67E-05 | 0.01985321 |
| OAS2 | -5.2259512 | 2.63E-05 | 0.01985321 |
| LIPA | -5.2390499 | 2.55E-05 | 0.01985321 |
| lnc-AKR1C2-4 | -5.2715599 | 2.35E-05 | 0.01945101 |
| CEP128 | -5.2902552 | 2.25E-05 | 0.01893835 |
| RANBP1 | -5.309042 | 2.14E-05 | 0.01844259 |
| GEN1 | -5.3442366 | 1.97E-05 | 0.01792336 |
| ISG15 | -5.3814042 | 1.79E-05 | 0.01710565 |
| CENPK | -5.4026396 | 1.70E-05 | 0.01664436 |
| HERC5 | -5.4531618 | 1.50E-05 | 0.01504377 |
| CFAP46 | -5.4672354 | 1.45E-05 | 0.01487917 |
| ZWINT | -5.4680537 | 1.45E-05 | 0.01487917 |
| C16orf59 | -5.4721796 | 1.44E-05 | 0.01487917 |
| TRIM21 | -5.4800265 | 1.41E-05 | 0.01487917 |
| EIF2AK2 | -5.4922925 | 1.37E-05 | 0.01487917 |
| C14orf80 | -5.5139064 | 1.30E-05 | 0.01487917 |
| SGOL2 | -5.5144236 | 1.29E-05 | 0.01487917 |
| UBE2T | -5.5196399 | 1.28E-05 | 0.01487917 |
| KRT31 | -5.5207033 | 1.28E-05 | 0.01487917 |
| KIF24 | -5.5230311 | 1.27E-05 | 0.01487917 |
| OAS3 | -5.5637772 | 1.15E-05 | 0.01487917 |
| NCAPG2 | -5.6124447 | 1.02E-05 | 0.01413028 |
| CENPQ | -5.6315909 | 9.72E-06 | 0.0139349 |
| IRF7 | -5.657621 | 9.13E-06 | 0.0139349 |
| HCP5 | -5.7027465 | 8.18E-06 | 0.01352019 |
| SCLT1 | -5.7828535 | 6.73E-06 | 0.01205741 |
| RAD51D | -5.8007947 | 6.44E-06 | 0.01204623 |
| PARPBP | -5.8077916 | 6.34E-06 | 0.01204623 |
| RLN2 | -5.8146149 | 6.23E-06 | 0.01204623 |
| lnc-PPP3CA-1 | -5.9036152 | 5.03E-06 | 0.01137252 |
| PARP9 | -5.9119544 | 4.93E-06 | 0.01137252 |
| OAS1 | -6.0081383 | 3.91E-06 | 0.01050061 |
| SEMA3D | -6.0444142 | 3.58E-06 | 0.01026679 |
| NMI | -6.0923474 | 3.19E-06 | 0.00980695 |
| WDHD1 | -6.1409343 | 2.84E-06 | 0.00940341 |
| CYP2J2 | -6.1567049 | 2.74E-06 | 0.00940341 |
| AMDHD1 | -6.227134 | 2.32E-06 | 0.00904983 |
| SPATA17 | -6.2869516 | 2.01E-06 | 0.00863676 |
| IRF9 | -6.4620826 | 1.33E-06 | 0.00634677 |
| HCG26 | -6.5782798 | 1.01E-06 | 0.00621495 |
| NEIL3 | -7.1989194 | 2.43E-07 | 0.00209066 |
| CSF2RA | -7.2770359 | 2.04E-07 | 0.00209066 |
| DDX60 | -7.3432477 | 1.76E-07 | 0.00209066 |
| CASC1 | -7.6902998 | 8.15E-08 | 0.00175111 |
| FTCDNL1 | -15.503294 | 1.06E-13 | 4.55E-09 |

**Table S3. Differentially expressed genes in *CDKN2A* mutated familial melanoma patients vs controls**

| **Gene ID** | **statistic** | **p-value** | **adj. p-value** |
| --- | --- | --- | --- |
| MROH7 | 6.86664109 | 5.67E-06 | 0.01625675 |
| TGIF1 | 6.60230447 | 8.87E-06 | 0.01892007 |
| GCM1 | 6.50058706 | 1.06E-05 | 0.01892007 |
| EIF3F | 6.32286536 | 1.44E-05 | 0.02060546 |
| SLC28A1 | 6.13172188 | 2.01E-05 | 0.02307345 |
| DUSP26 | 6.06180529 | 2.28E-05 | 0.02386052 |
| DHX30 | 6.01177483 | 2.49E-05 | 0.02386052 |
| FAM20C | 5.9435206 | 2.82E-05 | 0.02386052 |
| SHOX | 5.91066241 | 2.99E-05 | 0.02386052 |
| KCNQ4 | 5.74021761 | 4.08E-05 | 0.02580244 |
| USHBP1 | 5.7388414 | 4.09E-05 | 0.02580244 |
| LINC01341 | 5.66842429 | 4.65E-05 | 0.02580244 |
| LOC440028 | 5.61268235 | 5.15E-05 | 0.02662476 |
| FAM186B | 5.58913044 | 5.38E-05 | 0.02662476 |
| LOC100132368 | 5.58667839 | 5.40E-05 | 0.02662476 |
| RP9P | 5.44879031 | 6.98E-05 | 0.02856584 |
| JMJD6 | 5.43564562 | 7.16E-05 | 0.02856584 |
| LINC00106 | 5.429264 | 7.24E-05 | 0.02856584 |
| C14orf180 | 5.36203615 | 8.21E-05 | 0.02885733 |
| XLOC_l2_001687 | 5.30747713 | 9.10E-05 | 0.02987597 |
| TAF5L | 5.26085906 | 9.94E-05 | 0.03132635 |
| CITED2 | 5.23116493 | 1.05E-04 | 0.03206482 |
| lnc-AGPAT6-1 | 5.22377977 | 1.07E-04 | 0.03206482 |
| TMEM151A | 5.20548985 | 1.10E-04 | 0.03214238 |
| SLC25A3P1 | 5.18573444 | 1.15E-04 | 0.03214238 |
| LOC401557 | 5.18466935 | 1.15E-04 | 0.03214238 |
| CTSB | 5.18345024 | 1.15E-04 | 0.03214238 |
| LOC101059906 | 5.13427436 | 1.26E-04 | 0.03385996 |
| PCED1A | 5.10287422 | 1.34E-04 | 0.03483897 |
| lnc-GLIPR1-3 | 5.0975095 | 1.36E-04 | 0.03483897 |
| LOC100128882 | 5.09724765 | 1.36E-04 | 0.03483897 |
| KLRG2 | 5.02475708 | 1.56E-04 | 0.03648047 |
| HCCAT5 | 4.99666626 | 1.65E-04 | 0.03687457 |
| MGC57346-CRHR1 | 4.99237698 | 1.66E-04 | 0.03687457 |
| BET1L | 4.98453496 | 1.68E-04 | 0.03687457 |
| LOC101927910 | 4.95554075 | 1.78E-04 | 0.03790591 |
| REN | 4.92457068 | 1.89E-04 | 0.03911819 |
| LOC151484 | 4.92430567 | 1.89E-04 | 0.03911819 |
| C20orf62 | 4.86806256 | 2.11E-04 | 0.03982211 |
| SELM | 4.86038507 | 2.14E-04 | 0.03982211 |
| GPR161 | 4.85491823 | 2.16E-04 | 0.03982211 |
| CASS4 | 4.84244482 | 2.22E-04 | 0.03982211 |
| PHOSPHO1 | 4.82061757 | 2.31E-04 | 0.04048208 |
| SNORA71A | 4.78594087 | 2.48E-04 | 0.04086418 |
| LIMK1 | 4.78259698 | 2.49E-04 | 0.04086418 |
| GPR3 | 4.7759761 | 2.52E-04 | 0.04086418 |
| CACTIN | 4.76417829 | 2.58E-04 | 0.04086418 |
| GDF7 | 4.75987749 | 2.61E-04 | 0.04086418 |
| OSCAR | 4.75507133 | 2.63E-04 | 0.04086418 |
| GZMM | 4.75108526 | 2.65E-04 | 0.04086418 |
| SLC7A5P1 | 4.70713231 | 2.89E-04 | 0.04223621 |
| BCAR1 | 4.68513682 | 3.02E-04 | 0.04280273 |
| TLE1 | 4.68118023 | 3.04E-04 | 0.04280273 |
| C7orf61 | 4.6626816 | 3.15E-04 | 0.04280273 |
| CNOT3 | 4.65536003 | 3.20E-04 | 0.04295993 |
| CHIA | 4.64259519 | 3.28E-04 | 0.04320352 |
| ZNF333 | 4.57663618 | 3.73E-04 | 0.04559096 |
| LOC100133286 | 4.57026154 | 3.78E-04 | 0.04575803 |
| EPS8L3 | 4.56951958 | 3.79E-04 | 0.04575803 |
| HAPLN2 | 4.5682328 | 3.80E-04 | 0.04575803 |
| PROP1 | 4.56193961 | 3.84E-04 | 0.04575803 |
| lnc-CARHSP1-1 | 4.5605887 | 3.85E-04 | 0.04575803 |
| VPREB3 | 4.55844889 | 3.87E-04 | 0.04582518 |
| ORMDL3 | 4.55529498 | 3.89E-04 | 0.04594315 |
| AATK | 4.55297952 | 3.91E-04 | 0.04594315 |
| LINC01478 | 4.54240434 | 3.99E-04 | 0.0467812 |
| LINC01118 | 4.53700498 | 4.04E-04 | 0.0469039 |
| MPZ | 4.52678704 | 4.12E-04 | 0.04760388 |
| HEPH | 4.50337033 | 4.31E-04 | 0.04820531 |
| MGP | 4.50308685 | 4.32E-04 | 0.04820531 |
| RNA18S5 | 4.49575276 | 4.38E-04 | 0.04859824 |
| GNL1 | 4.49466245 | 4.39E-04 | 0.04859824 |
| SLC22A23 | 4.4750744 | 4.56E-04 | 0.04941666 |
| EFCAB11 | -4.4725991 | 4.59E-04 | 0.04953507 |
| ACLY | -4.4766087 | 4.55E-04 | 0.04941649 |
| MPRIP | -4.4772455 | 4.54E-04 | 0.04941649 |
| FANCD2 | -4.4783067 | 4.53E-04 | 0.04941649 |
| TGDS | -4.485963 | 4.47E-04 | 0.04922936 |
| BMP2K | -4.49377 | 4.40E-04 | 0.04859824 |
| TCF19 | -4.49822 | 4.36E-04 | 0.04854611 |
| HEMK1 | -4.5033478 | 4.31E-04 | 0.04820531 |
| GTF2H3 | -4.5034569 | 4.31E-04 | 0.04820531 |
| ALMS1P | -4.5057802 | 4.29E-04 | 0.04820531 |
| GSTCD | -4.5129971 | 4.23E-04 | 0.04806517 |
| E2F8 | -4.518444 | 4.19E-04 | 0.04788126 |
| SENP8 | -4.5205741 | 4.17E-04 | 0.04785697 |
| G2E3 | -4.5226586 | 4.15E-04 | 0.04785697 |
| LAMP3 | -4.5303417 | 4.09E-04 | 0.04739782 |
| LOC100288842 | -4.5396992 | 4.02E-04 | 0.0467812 |
| BTG4 | -4.554044 | 3.90E-04 | 0.04594315 |
| KBTBD3 | -4.5627651 | 3.84E-04 | 0.04575803 |
| ZBED8 | -4.5636121 | 3.83E-04 | 0.04575803 |
| CDC23 | -4.5638194 | 3.83E-04 | 0.04575803 |
| TWF1 | -4.5745973 | 3.75E-04 | 0.04564505 |
| LINC01510 | -4.5772879 | 3.73E-04 | 0.04559096 |
| ATE1-AS1 | -4.5786609 | 3.72E-04 | 0.04559096 |
| CFDP1 | -4.5797851 | 3.71E-04 | 0.04559096 |
| TRIT1 | -4.5803964 | 3.71E-04 | 0.04559096 |
| RAD54B | -4.5872107 | 3.66E-04 | 0.0454243 |
| GEN1 | -4.5883854 | 3.65E-04 | 0.0454243 |
| EXTL2 | -4.5914772 | 3.63E-04 | 0.04530572 |
| C17orf85 | -4.5946425 | 3.60E-04 | 0.04515521 |
| CPPED1 | -4.5958345 | 3.59E-04 | 0.04515521 |
| MPC1 | -4.5962307 | 3.59E-04 | 0.04515521 |
| KIF21A | -4.6035979 | 3.54E-04 | 0.04475687 |
| USP8 | -4.6054226 | 3.53E-04 | 0.04475687 |
| MRPL19 | -4.6139852 | 3.47E-04 | 0.04424099 |
| FAM72D | -4.6198607 | 3.43E-04 | 0.04386241 |
| FANCB | -4.6304223 | 3.36E-04 | 0.04334734 |
| SKA2 | -4.630501 | 3.36E-04 | 0.04334734 |
| FERMT1 | -4.6330082 | 3.34E-04 | 0.04334734 |
| DHTKD1 | -4.6360667 | 3.32E-04 | 0.04325852 |
| KDM4C | -4.6373861 | 3.31E-04 | 0.04325852 |
| VRK1 | -4.6376934 | 3.31E-04 | 0.04325852 |
| PRDM10 | -4.6413586 | 3.29E-04 | 0.04320352 |
| RBM26 | -4.6424085 | 3.28E-04 | 0.04320352 |
| HERC5 | -4.642967 | 3.28E-04 | 0.04320352 |
| SCEL | -4.6452291 | 3.26E-04 | 0.04320352 |
| ASB9 | -4.6536592 | 3.21E-04 | 0.04295993 |
| LTN1 | -4.6603062 | 3.17E-04 | 0.04280273 |
| PKP4 | -4.6606183 | 3.16E-04 | 0.04280273 |
| SIRT5 | -4.6615857 | 3.16E-04 | 0.04280273 |
| CPNE8 | -4.6620227 | 3.16E-04 | 0.04280273 |
| C16orf59 | -4.6642679 | 3.14E-04 | 0.04280273 |
| CREM | -4.6649374 | 3.14E-04 | 0.04280273 |
| MITD1 | -4.6656332 | 3.13E-04 | 0.04280273 |
| HLA-J | -4.6728565 | 3.09E-04 | 0.04280273 |
| GNG10 | -4.677113 | 3.06E-04 | 0.04280273 |
| LOC100132057 | -4.6775431 | 3.06E-04 | 0.04280273 |
| RAB27A | -4.6817668 | 3.04E-04 | 0.04280273 |
| LRIG2 | -4.6850584 | 3.02E-04 | 0.04280273 |
| KIF14 | -4.6902954 | 2.99E-04 | 0.04280273 |
| CCDC50 | -4.6914119 | 2.98E-04 | 0.04280273 |
| TAP1 | -4.6995603 | 2.93E-04 | 0.04257597 |
| lnc-PPP3CA-1 | -4.7021017 | 2.92E-04 | 0.0425081 |
| SMC2 | -4.7071106 | 2.89E-04 | 0.04223621 |
| CLTC | -4.707993 | 2.88E-04 | 0.04223621 |
| CMTR2 | -4.7146159 | 2.85E-04 | 0.04219406 |
| TRIM14 | -4.7154391 | 2.84E-04 | 0.04219406 |
| C5orf15 | -4.7185105 | 2.82E-04 | 0.04216438 |
| PLSCR1 | -4.7291602 | 2.77E-04 | 0.04158356 |
| NUDT16 | -4.7335901 | 2.74E-04 | 0.04151512 |
| TCEAL4 | -4.7365498 | 2.73E-04 | 0.04142137 |
| BLM | -4.7398077 | 2.71E-04 | 0.04130431 |
| EIF2AK2 | -4.7446628 | 2.68E-04 | 0.04105977 |
| TFCP2 | -4.7484475 | 2.66E-04 | 0.04090276 |
| BRIP1 | -4.7507615 | 2.65E-04 | 0.04086418 |
| LSM5 | -4.7523004 | 2.64E-04 | 0.04086418 |
| NUCKS1 | -4.7542239 | 2.63E-04 | 0.04086418 |
| NCAPD2 | -4.7598161 | 2.61E-04 | 0.04086418 |
| RHPN1-AS1 | -4.7624223 | 2.59E-04 | 0.04086418 |
| TRIM5 | -4.7625621 | 2.59E-04 | 0.04086418 |
| ARPC2 | -4.7671018 | 2.57E-04 | 0.04086418 |
| PPHLN1 | -4.7683638 | 2.56E-04 | 0.04086418 |
| CDC25C | -4.778122 | 2.51E-04 | 0.04086418 |
| TCAIM | -4.7828988 | 2.49E-04 | 0.04086418 |
| RGS18 | -4.7863387 | 2.47E-04 | 0.04086418 |
| FAM122B | -4.7873415 | 2.47E-04 | 0.04086418 |
| DNAAF3 | -4.7904376 | 2.45E-04 | 0.04086418 |
| HERC6 | -4.7972272 | 2.42E-04 | 0.04086418 |
| TTK | -4.7980093 | 2.42E-04 | 0.04086418 |
| MPHOSPH9 | -4.8054558 | 2.38E-04 | 0.04066404 |
| SEMA3D | -4.8085851 | 2.37E-04 | 0.04057801 |
| PKIB | -4.8112404 | 2.36E-04 | 0.04053024 |
| PRTFDC1 | -4.8118771 | 2.35E-04 | 0.04053024 |
| ZNF252P | -4.8149222 | 2.34E-04 | 0.04053024 |
| IFI44L | -4.8191741 | 2.32E-04 | 0.04048208 |
| ZNF480 | -4.8249296 | 2.29E-04 | 0.04043517 |
| TBC1D19 | -4.8332927 | 2.26E-04 | 0.03994654 |
| MMS22L | -4.8391486 | 2.23E-04 | 0.03982211 |
| CASP8 | -4.8428075 | 2.22E-04 | 0.03982211 |
| BCKDHB | -4.8468167 | 2.20E-04 | 0.03982211 |
| AIM2 | -4.8479042 | 2.19E-04 | 0.03982211 |
| CDNF | -4.849987 | 2.19E-04 | 0.03982211 |
| BRCA1 | -4.8520544 | 2.18E-04 | 0.03982211 |
| PKP4 | -4.8564741 | 2.16E-04 | 0.03982211 |
| CEP128 | -4.8589963 | 2.15E-04 | 0.03982211 |
| COMMD10 | -4.8590538 | 2.15E-04 | 0.03982211 |
| MOCS2 | -4.8603084 | 2.14E-04 | 0.03982211 |
| MTERF2 | -4.860749 | 2.14E-04 | 0.03982211 |
| METTL14 | -4.8692787 | 2.11E-04 | 0.03982211 |
| CEP295 | -4.8713141 | 2.10E-04 | 0.03982211 |
| CSF2RA | -4.8769873 | 2.07E-04 | 0.03982211 |
| PPP2R5E | -4.8794425 | 2.06E-04 | 0.03982211 |
| MRPS14 | -4.884928 | 2.04E-04 | 0.03982211 |
| TICRR | -4.8863851 | 2.04E-04 | 0.03982211 |
| MLLT4-AS1 | -4.8864866 | 2.04E-04 | 0.03982211 |
| KIAA0895 | -4.8925029 | 2.01E-04 | 0.03982211 |
| HLA-F | -4.89301 | 2.01E-04 | 0.03982211 |
| JRKL | -4.894311 | 2.01E-04 | 0.03982211 |
| HMGN5 | -4.9006776 | 1.98E-04 | 0.03982211 |
| IFITM4P | -4.9030459 | 1.97E-04 | 0.03982211 |
| CHM | -4.9065221 | 1.96E-04 | 0.03982211 |
| ZC2HC1C | -4.9125414 | 1.94E-04 | 0.03982211 |
| ACTR6 | -4.9392208 | 1.84E-04 | 0.03859187 |
| BEX5 | -4.9624187 | 1.76E-04 | 0.03779579 |
| FAM208A | -4.9713511 | 1.73E-04 | 0.03733696 |
| OAS1 | -4.98043 | 1.70E-04 | 0.03687457 |
| LIPA | -4.9805643 | 1.70E-04 | 0.03687457 |
| MRPL1 | -4.990945 | 1.66E-04 | 0.03687457 |
| DARS2 | -4.9954294 | 1.65E-04 | 0.03687457 |
| PLIN4 | -5.0067252 | 1.61E-04 | 0.03685749 |
| FAM161A | -5.02047 | 1.57E-04 | 0.03648701 |
| KIAA1107 | -5.0241231 | 1.56E-04 | 0.03648047 |
| CYP2J2 | -5.027391 | 1.55E-04 | 0.03648047 |
| PIGV | -5.0362053 | 1.53E-04 | 0.03623436 |
| YWHAZ | -5.037316 | 1.52E-04 | 0.03623436 |
| FAM111B | -5.0471995 | 1.49E-04 | 0.03607567 |
| MSL3P1 | -5.0601967 | 1.46E-04 | 0.03558735 |
| TMPO | -5.0609967 | 1.45E-04 | 0.03558735 |
| ECT2 | -5.0611705 | 1.45E-04 | 0.03558735 |
| STXBP5 | -5.0662369 | 1.44E-04 | 0.03558735 |
| MIPOL1 | -5.0671159 | 1.44E-04 | 0.03558735 |
| GLCE | -5.0809206 | 1.40E-04 | 0.03544996 |
| NCAPD3 | -5.1021028 | 1.34E-04 | 0.03483897 |
| APC | -5.1168061 | 1.31E-04 | 0.03448053 |
| TBC1D8B | -5.1324789 | 1.27E-04 | 0.03385996 |
| FAM107B | -5.1351562 | 1.26E-04 | 0.03385996 |
| TSGA10 | -5.1426441 | 1.24E-04 | 0.03385996 |
| SNX6 | -5.1475027 | 1.23E-04 | 0.03376163 |
| CENPK | -5.159618 | 1.20E-04 | 0.03341686 |
| TCF12 | -5.1900274 | 1.14E-04 | 0.03214238 |
| RAD51AP1 | -5.1957026 | 1.12E-04 | 0.03214238 |
| DSCAM | -5.1988736 | 1.12E-04 | 0.03214238 |
| ANP32E | -5.2086434 | 1.10E-04 | 0.03214238 |
| RPGRIP1L | -5.2152029 | 1.08E-04 | 0.03214238 |
| ATG10 | -5.2237446 | 1.07E-04 | 0.03206482 |
| RCC2 | -5.2245527 | 1.06E-04 | 0.03206482 |
| ATAD5 | -5.2258203 | 1.06E-04 | 0.03206482 |
| FKTN | -5.2445116 | 1.03E-04 | 0.03194426 |
| IRF9 | -5.2586721 | 9.98E-05 | 0.03132635 |
| CENPQ | -5.2762625 | 9.66E-05 | 0.03075041 |
| MCTP2 | -5.2939173 | 9.34E-05 | 0.03018923 |
| C1orf112 | -5.2950876 | 9.32E-05 | 0.03018923 |
| ME1 | -5.3111863 | 9.04E-05 | 0.02987597 |
| SUCLG2 | -5.3120429 | 9.03E-05 | 0.02987597 |
| LOC256880 | -5.3431228 | 8.51E-05 | 0.02904462 |
| PARPBP | -5.3476918 | 8.44E-05 | 0.02902647 |
| PRKDC | -5.3548338 | 8.33E-05 | 0.02887029 |
| PTPN13 | -5.3593813 | 8.26E-05 | 0.02885733 |
| CPEB3 | -5.3731651 | 8.05E-05 | 0.02882287 |
| EXOC4 | -5.3790366 | 7.96E-05 | 0.02874655 |
| LARP1B | -5.3791075 | 7.96E-05 | 0.02874655 |
| SRBD1 | -5.3837072 | 7.89E-05 | 0.02874655 |
| EDEM2 | -5.4008136 | 7.64E-05 | 0.02874655 |
| C2CD5 | -5.4041473 | 7.59E-05 | 0.02874655 |
| GLYCTK | -5.4101594 | 7.51E-05 | 0.02874655 |
| TRRAP | -5.4316732 | 7.21E-05 | 0.02856584 |
| S100A10 | -5.4365155 | 7.14E-05 | 0.02856584 |
| UEVLD | -5.4403974 | 7.09E-05 | 0.02856584 |
| BTBD8 | -5.4432552 | 7.06E-05 | 0.02856584 |
| NMI | -5.4539202 | 6.92E-05 | 0.02856584 |
| NCAPG2 | -5.479234 | 6.60E-05 | 0.02808315 |
| PARP9 | -5.48792 | 6.49E-05 | 0.02790878 |
| NUP205 | -5.4885719 | 6.48E-05 | 0.02790878 |
| TCF20 | -5.4919906 | 6.44E-05 | 0.02790878 |
| CLUAP1 | -5.4921588 | 6.44E-05 | 0.02790878 |
| ZNF518A | -5.4972631 | 6.38E-05 | 0.02790878 |
| UBE2T | -5.5163622 | 6.16E-05 | 0.02790878 |
| OIP5-AS1 | -5.5363925 | 5.93E-05 | 0.02790878 |
| lnc-SNURF-3 | -5.5821856 | 5.45E-05 | 0.02662476 |
| NEIL3 | -5.5827206 | 5.44E-05 | 0.02662476 |
| GSAP | -5.583014 | 5.44E-05 | 0.02662476 |
| WDHD1 | -5.587623 | 5.40E-05 | 0.02662476 |
| IFT88 | -5.6646205 | 4.68E-05 | 0.02580244 |
| TTC13 | -5.6716889 | 4.62E-05 | 0.02580244 |
| ZSCAN20 | -5.6763441 | 4.58E-05 | 0.02580244 |
| CASD1 | -5.6857226 | 4.50E-05 | 0.02580244 |
| SLC10A7 | -5.6865145 | 4.50E-05 | 0.02580244 |
| TMEM194A | -5.713133 | 4.28E-05 | 0.02580244 |
| HCG26 | -5.7198299 | 4.23E-05 | 0.02580244 |
| lnc-INTS9-1 | -5.7316192 | 4.14E-05 | 0.02580244 |
| FANCA | -5.7520231 | 3.99E-05 | 0.02580244 |
| RAD51D | -5.7650667 | 3.89E-05 | 0.02580244 |
| lnc-ITGA2-1 | -5.826373 | 3.48E-05 | 0.02455037 |
| EHHADH | -5.8334766 | 3.44E-05 | 0.02455037 |
| lnc-AKR1C2-4 | -5.8671198 | 3.24E-05 | 0.02386052 |
| APITD1 | -5.8690469 | 3.22E-05 | 0.02386052 |
| HLA-G | -5.8698302 | 3.22E-05 | 0.02386052 |
| RNF180 | -5.8708997 | 3.21E-05 | 0.02386052 |
| RANBP1 | -5.8717388 | 3.21E-05 | 0.02386052 |
| RLN2 | -5.9192971 | 2.94E-05 | 0.02386052 |
| ERI2 | -5.9230865 | 2.92E-05 | 0.02386052 |
| FAM178A | -5.9437413 | 2.82E-05 | 0.02386052 |
| CBL | -5.9693521 | 2.69E-05 | 0.02386052 |
| SGOL2 | -5.9973564 | 2.56E-05 | 0.02386052 |
| SPATA17 | -6.0072573 | 2.51E-05 | 0.02386052 |
| AMDHD1 | -6.0605358 | 2.29E-05 | 0.02386052 |
| C18orf54 | -6.1098415 | 2.09E-05 | 0.02307345 |
| KIAA1841 | -6.1238112 | 2.04E-05 | 0.02307345 |
| RTKN2 | -6.129118 | 2.02E-05 | 0.02307345 |
| HCP5 | -6.1862416 | 1.83E-05 | 0.02307345 |
| NEB | -6.2043542 | 1.77E-05 | 0.02306622 |
| ZCWPW2 | -6.2050957 | 1.77E-05 | 0.02306622 |
| KLHL12 | -6.2321042 | 1.69E-05 | 0.02306622 |
| POC1B | -6.32414 | 1.43E-05 | 0.02060546 |
| FGFR1OP | -6.374577 | 1.31E-05 | 0.02017295 |
| KDM3B | -6.3817718 | 1.30E-05 | 0.02017295 |
| TSKS | -6.3887785 | 1.28E-05 | 0.02017295 |
| NUP107 | -6.4081301 | 1.24E-05 | 0.02017295 |
| CASP8 | -6.5102949 | 1.04E-05 | 0.01892007 |
| SCLT1 | -6.5274812 | 1.01E-05 | 0.01892007 |
| KIF24 | -6.5341446 | 9.97E-06 | 0.01892007 |
| FAM129A | -6.5501553 | 9.70E-06 | 0.01892007 |
| CATSPER3 | -6.7264441 | 7.18E-06 | 0.01715244 |
| MPV17L | -6.7361088 | 7.06E-06 | 0.01715244 |
| DDX60 | -6.7707387 | 6.66E-06 | 0.01715244 |
| TRMT10A | -6.9108158 | 5.27E-06 | 0.01617889 |
| SASS6 | -6.9650493 | 4.81E-06 | 0.01592003 |
| CASC1 | -6.9826702 | 4.68E-06 | 0.01592003 |
| ENTPD1-AS1 | -7.0061256 | 4.50E-06 | 0.01592003 |
| TMOD3 | -7.0160285 | 4.42E-06 | 0.01592003 |
| POLD3 | -7.1910731 | 3.32E-06 | 0.01592003 |
| DZIP3 | -7.3173686 | 2.70E-06 | 0.01592003 |
| DUSP19 | -7.4321373 | 2.25E-06 | 0.01592003 |
| MDM1 | -8.3955498 | 5.12E-07 | 0.00439847 |
| C6orf211 | -8.8993384 | 2.47E-07 | 0.00265027 |
| FTCDNL1 | -12.121131 | 4.27E-09 | 1.83E-04 |

**Table S4. Differentially expressed genes in *CDKN2A* wild-type familial melanoma patients vs controls**

| **Gene ID** | **statistic** | **p-value** | **adj. p-value** |
| --- | --- | --- | --- |
| HEPH | 6.75261751 | 7.20E-06 | 0.0196353 |
| CHRM3 | 5.68061982 | 4.71E-05 | 0.04160728 |
| HLA-J | -5.6505215 | 4.97E-05 | 0.04191391 |
| IFI6 | -5.6653729 | 4.84E-05 | 0.04160728 |
| PSMB8 | -5.66823 | 4.81E-05 | 0.04160728 |
| MX2 | -5.6901518 | 4.62E-05 | 0.04160728 |
| TMPRSS11D | -5.7112867 | 4.45E-05 | 0.04159274 |
| HOXA9 | -5.7209522 | 4.37E-05 | 0.04159274 |
| RSAD2 | -5.8123518 | 3.70E-05 | 0.03620081 |
| HSH2D | -5.8152029 | 3.69E-05 | 0.03620081 |
| CCDC153 | -5.8617215 | 3.39E-05 | 0.03469413 |
| HOXA7 | -5.9004682 | 3.16E-05 | 0.03314951 |
| HCG26 | -5.9847004 | 2.72E-05 | 0.02997487 |
| EIF2AK2 | -6.0268329 | 2.52E-05 | 0.02854074 |
| TRIM36 | -6.040527 | 2.46E-05 | 0.02854074 |
| SAMD13 | -6.0621604 | 2.37E-05 | 0.02829542 |
| LYPD6 | -6.0881913 | 2.26E-05 | 0.02779291 |
| NMI | -6.1130358 | 2.17E-05 | 0.02738331 |
| IRF9 | -6.1247665 | 2.12E-05 | 0.02738331 |
| BCCIP | -6.1248533 | 2.12E-05 | 0.02738331 |
| EIF2AK2 | -6.1445372 | 2.05E-05 | 0.02738331 |
| OAS2 | -6.1479213 | 2.04E-05 | 0.02738331 |
| HERC5 | -6.1952052 | 1.87E-05 | 0.02738331 |
| IFI44 | -6.3449758 | 1.44E-05 | 0.02307556 |
| OASL | -6.3926374 | 1.33E-05 | 0.02285461 |
| PARP9 | -6.3999317 | 1.31E-05 | 0.02285461 |
| IFIT1 | -6.4117991 | 1.29E-05 | 0.02285461 |
| lnc-PPP3CA-1 | -6.4492549 | 1.21E-05 | 0.02285461 |
| TRIM21 | -6.5160501 | 1.08E-05 | 0.02200899 |
| USP41 | -6.5195083 | 1.07E-05 | 0.02200899 |
| IRF7 | -6.5751557 | 9.72E-06 | 0.02199284 |
| SEMA3D | -6.5936957 | 9.42E-06 | 0.02199284 |
| HOXA6 | -6.6492804 | 8.57E-06 | 0.02167553 |
| USP18 | -6.8448552 | 6.17E-06 | 0.0196353 |
| CSF2RA | -6.9703328 | 5.01E-06 | 0.01796528 |
| CASC1 | -6.9930616 | 4.83E-06 | 0.01796528 |
| ISG15 | -7.0236369 | 4.59E-06 | 0.01796528 |
| CYP2J2 | -7.0670728 | 4.28E-06 | 0.01796528 |
| OAS1 | -7.3812478 | 2.58E-06 | 0.01384602 |
| STAT1 | -7.6715002 | 1.63E-06 | 0.01001909 |
| OAS2 | -7.7884269 | 1.36E-06 | 0.00975302 |
| CMPK2 | -7.9071571 | 1.13E-06 | 0.00975302 |
| OAS3 | -8.2389907 | 6.88E-07 | 0.00739844 |
| DDX60 | -8.3601374 | 5.75E-07 | 0.00739844 |
| FTCDNL1 | -12.167535 | 4.49E-09 | 1.93E-04 |

**Table S5. Significant KEEGs overrepresented in downregulated genes in familial melanoma patients vs controls**

| **KEGG pathway** | **Adj. P*** |
| --- | --- |
| Fanconi anemia pathway (KEGG:03460) | 1.718E-06 |
| Homologous recombination (KEGG:03440) | 3.937E-06 |
| Mismatch repair (KEGG:03430) | 3.945E-03 |
| DNA replication (KEGG:03030) | 2.397E-02 |
| Herpes simplex infection (KEGG:05168) | 5.463E-08 |
| Epstein-Barr virus infection (KEGG:05169) | 1.326E-05 |
| Measles (KEGG:05162) | 1.884E-04 |
| Influenza A (KEGG:05164) | 1.556E-03 |
| Hepatitis C (KEGG:05160) | 8.099E-04 |
| NOD-like receptor signaling pathway (KEGG:04621) | 1.021E-02 |
| Kaposi sarcoma-associated herpesvirus infection (KEGG:05167) | 2.073E-02 |

*Bonferroni correction was used

**Table S6. Top 20 (of 338) significant GOs overrepresented in downregulated genes in familial melanoma patients vs controls**

| **Gene Ontology (GO) term** | **P-value** | **Adj. P-value** |
| --- | --- | --- |
| response to type I interferon(GO:0034340) | 1.91E-23 | 5.53E-20 |
| cellular response to type I interferon(GO:0071357) | 1.75E-23 | 5.53E-20 |
| type I interferon signaling pathway(GO:0060337) | 1.75E-23 | 5.53E-20 |
| chromosome segregation(GO:0007059) | 1.84E-22 | 3.98E-19 |
| defense response to virus(GO:0051607) | 5.07E-19 | 8.81E-16 |
| DNA-dependent DNA replication(GO:0006261) | 1.87E-17 | 2.70E-14 |
| double-strand break repair(GO:0006302) | 2.18E-17 | 2.70E-14 |
| double-strand break repair via homologous recombination(GO:0000724) | 7.92E-16 | 8.60E-13 |
| recombinational repair(GO:0000725) | 9.54E-16 | 9.21E-13 |
| regulation of microtubule cytoskeleton organization(GO:0070507) | 4.32E-15 | 3.75E-12 |
| regulation of microtubule-based process(GO:0032886) | 2.27E-14 | 1.79E-11 |
| meiotic cell cycle(GO:0051321) | 1.30E-13 | 9.42E-11 |
| mitotic recombination(GO:0006312) | 3.35E-13 | 2.24E-10 |
| regulation of mitotic nuclear division(GO:0007088) | 4.83E-13 | 2.99E-10 |
| meiotic nuclear division(GO:0007126) | 5.49E-13 | 3.18E-10 |
| meiotic cell cycle process(GO:1903046) | 9.40E-13 | 5.10E-10 |
| histone exchange(GO:0043486) | 1.38E-12 | 6.65E-10 |
| regulation of nuclear division(GO:0051783) | 1.36E-12 | 6.65E-10 |
| response to interferon-gamma(GO:0034341) | 4.28E-12 | 1.95E-09 |
| interferon-gamma-mediated signaling pathway(GO:0060333) | 6.29E-12 | 2.73E-09 |

**Table S7. DEGs identified in our study and previous association with aging**

| **GENE** | **Comparisons with statistically significance** | **Associated with skin aging in previous studies** | **References** |
| --- | --- | --- | --- |
| *AATK* | *CDKN2A* carriers vs. controls | YES | 17 |
| *ACLY* | All melanoma patients vs. controls  *CDKN2A* carriers vs. controls | YES | 17, 18 |
| *ACTR3* | All melanoma patients vs. controls | NO |  |
| *ACTR6* | *CDKN2A* carriers vs. controls | NO |  |
| *ADAMTSL3* | All melanoma patients vs. controls | YES | 17, 18 |
| *AIM2* | All melanoma patients vs. controls  *CDKN2A* carriers vs. controls | NO |  |
| *ALMS1P* | *CDKN2A* carriers vs. controls | NO |  |
| *AMDHD1* | All melanoma patients vs. controls  *CDKN2A* carriers vs. controls | NO |  |
| *ANP32E* | All melanoma patients vs. controls  *CDKN2A* carriers vs. controls | NO |  |
| *APC* | All melanoma patients vs. controls  *CDKN2A* carriers vs. controls | NO |  |
| *APITD1* | All melanoma patients vs. controls  *CDKN2A* carriers vs. controls | YES | 18 |
| *ARPC2* | All melanoma patients vs. controls  *CDKN2A* carriers vs. controls | YES | 17, 18 |
| *ASB9* | *CDKN2A* carriers vs. controls | YES | 17, 18 |
| *ASPG* | All melanoma patients vs. controls | NO |  |
| *ASPM* | All melanoma patients vs. controls | YES | 17, 18 |
| *ATAD5* | All melanoma patients vs. controls  *CDKN2A* carriers vs. controls | NO |  |
| *ATE1-AS1* | *CDKN2A* carriers vs. controls | NO |  |
| *ATG10* | *CDKN2A* carriers vs. controls | YES | 18 |
| *AURKA* | All melanoma patients vs. controls | NO |  |
| *AURKB* | All melanoma patients vs. controls | NO |  |
| *BCAR1* | *CDKN2A* carriers vs. controls | NO |  |
| *BCCIP* | *CDKN2A* WT vs. controls | NO |  |
| *BCKDHB* | *CDKN2A* carriers vs. controls | YES | 17, 18 |
| *BEND6* | All melanoma patients vs. controls | YES | 18 |
| *BET1L* | *CDKN2A* carriers vs. controls | NO |  |
| *BEX5* | *CDKN2A* carriers vs. controls | NO |  |
| *BLM* | All melanoma patients vs. controls  *CDKN2A* carriers vs. controls | NO |  |
| *BMP2K* | *CDKN2A* carriers vs. controls | NO |  |
| *BORA* | All melanoma patients vs. controls | NO |  |
| *BRCA1* | All melanoma patients vs. controls  *CDKN2A* carriers vs. controls | NO |  |
| *BRCA2* | All melanoma patients vs. controls | YES | 18 |
| *BRIP1* | All melanoma patients vs. controls  *CDKN2A* carriers vs. controls | NO |  |
| *BTBD8* | *CDKN2A* carriers vs. controls | NO |  |
| *BTG4* | *CDKN2A* carriers vs. controls | NO |  |
| *C14orf180* | All melanoma patients vs. controls  *CDKN2A* carriers vs. controls | NO |  |
| *C14orf80* | All melanoma patients vs. controls | NO |  |
| *C16orf59* | All melanoma patients vs. controls  *CDKN2A* carriers vs. controls | NO |  |
| *C17orf85* | *CDKN2A* carriers vs. controls | YES | 18 |
| *C18orf54* | *CDKN2A* carriers vs. controls | NO |  |
| *C1orf112* | All melanoma patients vs. controls  *CDKN2A* carriers vs. controls | NO |  |
| *C20orf62* | *CDKN2A* carriers vs. controls | NO |  |
| *C2CD5* | All melanoma patients vs. controls  *CDKN2A* carriers vs. controls | NO |  |
| *C5orf15* | *CDKN2A* carriers vs. controls | NO |  |
| *C6orf211* | All melanoma patients vs. controls  *CDKN2A* carriers vs. controls | NO |  |
| *C7orf61* | *CDKN2A* carriers vs. controls | NO |  |
| *C8orf48* | All melanoma patients vs. controls | NO |  |
| *CACTIN* | *CDKN2A* carriers vs. controls | NO |  |
| *CASC1* | All melanoma patients vs. controls  *CDKN2A* carriers vs. controls  *CDKN2A* WT vs. controls | YES | 17, 18 |
| *CASC5* | All melanoma patients vs. controls | NO |  |
| *CASD1* | *CDKN2A* carriers vs. controls | NO |  |
| *CASP8* | All melanoma patients vs. controls  *CDKN2A* carriers vs. controls | NO |  |
| *CASS4* | *CDKN2A* carriers vs. controls | NO |  |
| *CATSPER3* | All melanoma patients vs. controls  *CDKN2A* carriers vs. controls | NO |  |
| *CBL* | *CDKN2A* carriers vs. controls | NO |  |
| *CCDC153* | *CDKN2A* WT vs. controls | NO |  |
| *CCDC50* | *CDKN2A* carriers vs. controls | YES | 18 |
| *CCDC88C* | All melanoma patients vs. controls | NO |  |
| *CDC23* | *CDKN2A* carriers vs. controls | NO |  |
| *CDC25C* | All melanoma patients vs. controls  *CDKN2A* carriers vs. controls | YES | 17, 18 |
| *CDNF* | *CDKN2A* carriers vs. controls | NO |  |
| *CENPA* | All melanoma patients vs. controls | NO |  |
| *CENPE* | All melanoma patients vs. controls | NO |  |
| *CENPI* | All melanoma patients vs. controls | NO |  |
| *CENPK* | All melanoma patients vs. controls  *CDKN2A* carriers vs. controls | NO |  |
| *CENPQ* | All melanoma patients vs. controls  *CDKN2A* carriers vs. controls | NO |  |
| *CEP128* | All melanoma patients vs. controls  *CDKN2A* carriers vs. controls | NO |  |
| *CEP295* | *CDKN2A* carriers vs. controls | NO |  |
| *CFAP46* | All melanoma patients vs. controls | NO |  |
| *CFDP1* | *CDKN2A* carriers vs. controls | NO |  |
| *CHIA* | *CDKN2A* carriers vs. controls | NO |  |
| *CHM* | *CDKN2A* carriers vs. controls | NO |  |
| *CHRM3* | *CDKN2A* WT vs. controls | NO |  |
| *CITED2* | *CDKN2A* carriers vs. controls | NO |  |
| *CKAP2* | All melanoma patients vs. controls | NO |  |
| *CLSPN* | All melanoma patients vs. controls | NO |  |
| *CLTC* | *CDKN2A* carriers vs. controls | NO |  |
| *CLUAP1* | *CDKN2A* carriers vs. controls | NO |  |
| *CMPK2* | All melanoma patients vs. controls  *CDKN2A* WT vs. controls | NO |  |
| *CMTR2* | *CDKN2A* carriers vs. controls | NO |  |
| *CNOT3* | *CDKN2A* carriers vs. controls | NO |  |
| *COMMD10* | *CDKN2A* carriers vs. controls | NO |  |
| *COMMD8* | All melanoma patients vs. controls | NO |  |
| *CPEB3* | *CDKN2A* carriers vs. controls | NO |  |
| *CPNE8* | *CDKN2A* carriers vs. controls | NO |  |
| *CPPED1* | *CDKN2A* carriers vs. controls | NO |  |
| *CREM* | *CDKN2A* carriers vs. controls | NO |  |
| *CSE1L* | All melanoma patients vs. controls | NO |  |
| *CSF2RA* | All melanoma patients vs. controls  *CDKN2A* carriers vs. controls  *CDKN2A* WT vs. controls | NO |  |
| *CTSB* | All melanoma patients vs. controls  *CDKN2A* carriers vs. controls | NO |  |
| *CYP2J2* | All melanoma patients vs. controls  *CDKN2A* carriers vs. controls  *CDKN2A* WT vs. controls | NO |  |
| *DARS2* | *CDKN2A* carriers vs. controls | NO |  |
| *DDIAS* | All melanoma patients vs. controls | NO |  |
| *DDX60* | All melanoma patients vs. controls  *CDKN2A* carriers vs. controls  *CDKN2A* WT vs. controls | NO |  |
| *DHTKD1* | All melanoma patients vs. controls  *CDKN2A* carriers vs. controls | NO |  |
| *DHX30* | *CDKN2A* carriers vs. controls | NO |  |
| *DMC1* | All melanoma patients vs. controls | NO |  |
| *DNAAF3* | All melanoma patients vs. controls  *CDKN2A* carriers vs. controls | NO |  |
| *DSCAM* | All melanoma patients vs. controls  *CDKN2A* carriers vs. controls | NO |  |
| *DSCC1* | All melanoma patients vs. controls | NO |  |
| *DUSP19* | All melanoma patients vs. controls  *CDKN2A* carriers vs. controls | NO |  |
| *DUSP26* | *CDKN2A* carriers vs. controls | NO |  |
| *DZIP3* | *CDKN2A* carriers vs. controls | NO |  |
| *E2F8* | *CDKN2A* carriers vs. controls | NO |  |
| *ECT2* | All melanoma patients vs. controls  *CDKN2A* carriers vs. controls | YES | 18 |
| *EDEM2* | *CDKN2A* carriers vs. controls | NO |  |
| *EFCAB11* | All melanoma patients vs. controls  *CDKN2A* carriers vs. controls | NO |  |
| *EHHADH* | *CDKN2A* carriers vs. controls | NO |  |
| *EIF2AK2* | All melanoma patients vs. controls  *CDKN2A* carriers vs. controls  *CDKN2A* WT vs. controls | YES | 18 |
| *EIF3F* | *CDKN2A* carriers vs. controls | NO |  |
| *EMP1* | All melanoma patients vs. controls | YES | 18 |
| *ENTPD1-AS1* | *CDKN2A* carriers vs. controls | NO |  |
| *EPS8L3* | *CDKN2A* carriers vs. controls | NO |  |
| *ERI2* | All melanoma patients vs. controls  *CDKN2A* carriers vs. controls | NO |  |
| *ESCO2* | All melanoma patients vs. controls | NO |  |
| *EXO1* | All melanoma patients vs. controls | NO |  |
| *EXOC4* | *CDKN2A* carriers vs. controls | YES | 18 |
| *EXTL2* | *CDKN2A* carriers vs. controls | YES | 18 |
| *FAM107B* | *CDKN2A* carriers vs. controls | NO |  |
| *FAM111B* | All melanoma patients vs. controls  *CDKN2A* carriers vs. controls | NO |  |
| *FAM122B* | *CDKN2A* carriers vs. controls | NO |  |
| *FAM129A* | All melanoma patients vs. controls  *CDKN2A* carriers vs. controls | YES | 18 |
| *FAM161A* | *CDKN2A* carriers vs. controls | NO |  |
| *FAM178A* | *CDKN2A* carriers vs. controls | NO |  |
| *FAM186B* | *CDKN2A* carriers vs. controls | NO |  |
| *FAM208A* | *CDKN2A* carriers vs. controls | NO |  |
| *FAM20C* | *CDKN2A* carriers vs. controls | NO |  |
| *FAM72D* | All melanoma patients vs. controls  *CDKN2A* carriers vs. controls | NO |  |
| *FANCA* | All melanoma patients vs. controls  *CDKN2A* carriers vs. controls | NO |  |
| *FANCB* | All melanoma patients vs. controls  *CDKN2A* carriers vs. controls | YES | 18 |
| *FANCD2* | *CDKN2A* carriers vs. controls | YES | 17, 18 |
| *FANCI* | All melanoma patients vs. controls | NO |  |
| *FERMT1* | All melanoma patients vs. controls  *CDKN2A* carriers vs. controls | YES | 18 |
| *FGFR1OP* | All melanoma patients vs. controls  *CDKN2A* carriers vs. controls | NO |  |
| *FKTN* | *CDKN2A* carriers vs. controls | NO |  |
| *FMO5* | All melanoma patients vs. controls | NO |  |
| *FOLH1* | All melanoma patients vs. controls | NO |  |
| *FOLH1B* | All melanoma patients vs. controls | NO |  |
| *FTCDNL1* | All melanoma patients vs. controls  *CDKN2A* carriers vs. controls  *CDKN2A* WT vs. controls | NO |  |
| *G2E3* | *CDKN2A* carriers vs. controls | NO |  |
| *GCM1* | *CDKN2A* carriers vs. controls | NO |  |
| *GDF7* | *CDKN2A* carriers vs. controls | NO |  |
| *GEN1* | All melanoma patients vs. controls  *CDKN2A* carriers vs. controls | NO |  |
| *GINS2* | All melanoma patients vs. controls | NO |  |
| *GLCE* | *CDKN2A* carriers vs. controls | YES | 18 |
| *GLYCTK* | *CDKN2A* carriers vs. controls | YES | 18 |
| *GNG10* | *CDKN2A* carriers vs. controls | NO |  |
| *GNL1* | *CDKN2A* carriers vs. controls | NO |  |
| *GP6* | All melanoma patients vs. controls | NO |  |
| *GPC6* | All melanoma patients vs. controls | NO |  |
| *GPR161* | *CDKN2A* carriers vs. controls | NO |  |
| *GPR3* | *CDKN2A* carriers vs. controls | NO |  |
| *GSAP* | All melanoma patients vs. controls  *CDKN2A* carriers vs. controls | NO |  |
| *GSTCD* | *CDKN2A* carriers vs. controls | YES | 18 |
| *GTF2H3* | *CDKN2A* carriers vs. controls | NO |  |
| *GZMM* | *CDKN2A* carriers vs. controls | NO |  |
| *HAPLN2* | *CDKN2A* carriers vs. controls | NO |  |
| *HCCAT5* | *CDKN2A* carriers vs. controls | NO |  |
| *HCG26* | All melanoma patients vs. controls  *CDKN2A* carriers vs. controls  *CDKN2A* WT vs. controls | NO |  |
| *HCP5* | All melanoma patients vs. controls  *CDKN2A* carriers vs. controls | YES | 17, 18 |
| *HEMK1* | *CDKN2A* carriers vs. controls | NO |  |
| *HEPH* | All melanoma patients vs. controls  *CDKN2A* carriers vs. controls  *CDKN2A* WT vs. controls | NO |  |
| *HERC5* | All melanoma patients vs. controls  *CDKN2A* carriers vs. controls  *CDKN2A* WT vs. controls | NO |  |
| *HERC6* | All melanoma patients vs. controls  *CDKN2A* carriers vs. controls | NO |  |
| *HLA-F* | All melanoma patients vs. controls  *CDKN2A* carriers vs. controls | YES | 17, 18 |
| *HLA-G* | All melanoma patients vs. controls  *CDKN2A* carriers vs. controls | NO |  |
| *HLA-J* | All melanoma patients vs. controls  *CDKN2A* carriers vs. controls  *CDKN2A* WT vs. controls | NO |  |
| *HMGN5* | All melanoma patients vs. controls  *CDKN2A* carriers vs. controls | NO |  |
| *HN1* | All melanoma patients vs. controls | NO |  |
| *HOXA6* | *CDKN2A* WT vs. controls | YES | 18 |
| *HOXA7* | All melanoma patients vs. controls  *CDKN2A* WT vs. controls | NO |  |
| *HOXA9* | *CDKN2A* WT vs. controls | YES | 17, 18 |
| *HSH2D* | *CDKN2A* WT vs. controls | NO |  |
| *HSPG2* | All melanoma patients vs. controls | NO |  |
| *ICA1L* | All melanoma patients vs. controls | NO |  |
| *IFI44* | All melanoma patients vs. controls  *CDKN2A* WT vs. controls | NO |  |
| *IFI44L* | All melanoma patients vs. controls  *CDKN2A* carriers vs. controls | NO |  |
| *IFI6* | All melanoma patients vs. controls  *CDKN2A* WT vs. controls | NO |  |
| *IFIH1* | All melanoma patients vs. controls | NO |  |
| *IFIT1* | All melanoma patients vs. controls  *CDKN2A* WT vs. controls | NO |  |
| *IFITM4P* | *CDKN2A* carriers vs. controls | NO |  |
| *IFT88* | *CDKN2A* carriers vs. controls | YES | 18 |
| *IGF2BP3* | All melanoma patients vs. controls | NO |  |
| *IL15RA* | All melanoma patients vs. controls | NO |  |
| *IQCH* | All melanoma patients vs. controls | NO |  |
| *IRF7* | All melanoma patients vs. controls  *CDKN2A* WT vs. controls | NO |  |
| *IRF9* | All melanoma patients vs. controls  *CDKN2A* carriers vs. controls  *CDKN2A* WT vs. controls | NO |  |
| *IRX1* | All melanoma patients vs. controls | NO |  |
| *ISG15* | All melanoma patients vs. controls  *CDKN2A* WT vs. controls | NO |  |
| *JAK2* | All melanoma patients vs. controls | YES | 17, 18 |
| *JMJD6* | *CDKN2A* carriers vs. controls | NO |  |
| *JRKL* | *CDKN2A* carriers vs. controls | NO |  |
| *KBTBD3* | *CDKN2A* carriers vs. controls | NO |  |
| *KCNQ4* | *CDKN2A* carriers vs. controls | NO |  |
| *KDM3B* | *CDKN2A* carriers vs. controls | NO |  |
| *KDM4C* | *CDKN2A* carriers vs. controls | NO |  |
| *KIAA0895* | *CDKN2A* carriers vs. controls | NO |  |
| *KIAA1107* | *CDKN2A* carriers vs. controls | NO |  |
| *KIAA1841* | *CDKN2A* carriers vs. controls | NO |  |
| *KIF14* | All melanoma patients vs. controls  *CDKN2A* carriers vs. controls | NO |  |
| *KIF20B* | All melanoma patients vs. controls | YES | 17, 18 |
| *KIF21A* | *CDKN2A* carriers vs. controls | NO |  |
| *KIF24* | All melanoma patients vs. controls  *CDKN2A* carriers vs. controls | NO |  |
| *KLHL12* | All melanoma patients vs. controls  *CDKN2A* carriers vs. controls | YES | 17, 18 |
| *KLRG2* | All melanoma patients vs. controls  *CDKN2A* carriers vs. controls | YES | 17, 18 |
| *KRT31* | All melanoma patients vs. controls | NO |  |
| *LAMP2* | All melanoma patients vs. controls | YES | 17, 18 |
| *LAMP3* | All melanoma patients vs. controls  *CDKN2A* carriers vs. controls | NO |  |
| *LAP3* | All melanoma patients vs. controls | NO |  |
| *LARP1B* | *CDKN2A* carriers vs. controls | NO |  |
| *LIMK1* | *CDKN2A* carriers vs. controls | YES | 18 |
| *LINC00106* | *CDKN2A* carriers vs. controls | NO |  |
| *LINC01118* | *CDKN2A* carriers vs. controls | NO |  |
| *LINC01133* | All melanoma patients vs. controls | NO |  |
| *LINC01341* | *CDKN2A* carriers vs. controls | NO |  |
| *LINC01478* | *CDKN2A* carriers vs. controls | NO |  |
| *LINC01510* | All melanoma patients vs. controls  *CDKN2A* carriers vs. controls | NO |  |
| *LINGO2* | All melanoma patients vs. controls | YES | 17, 18 |
| *LIPA* | All melanoma patients vs. controls  *CDKN2A* carriers vs. controls | YES | 18 |
| *LMF1* | All melanoma patients vs. controls | NO |  |
| *LMX1B* | All melanoma patients vs. controls | NO |  |
| *lnc-AGPAT6-1* | *CDKN2A* carriers vs. controls | NO |  |
| *lnc-AKR1C2-4* | All melanoma patients vs. controls  *CDKN2A* carriers vs. controls | NO |  |
| *lnc-CARHSP1-1* | *CDKN2A* carriers vs. controls | NO |  |
| *lnc-GLIPR1-3* | *CDKN2A* carriers vs. controls | NO |  |
| *lnc-INTS9-1* | *CDKN2A* carriers vs. controls | NO |  |
| *lnc-ITGA2-1* | All melanoma patients vs. controls  *CDKN2A* carriers vs. controls | NO |  |
| *lnc-PPP3CA-1* | All melanoma patients vs. controls  *CDKN2A* carriers vs. controls  *CDKN2A* WT vs. controls | NO |  |
| *lnc-SNURF-3* | *CDKN2A* carriers vs. controls | NO |  |
| *LOC100128882* | *CDKN2A* carriers vs. controls | NO |  |
| *LOC100132057* | *CDKN2A* carriers vs. controls | NO |  |
| *LOC100132368* | *CDKN2A* carriers vs. controls | NO |  |
| *LOC100133286* | *CDKN2A* carriers vs. controls | NO |  |
| *LOC100288842* | *CDKN2A* carriers vs. controls | NO |  |
| *LOC101059906* | *CDKN2A* carriers vs. controls | NO |  |
| *LOC101927910* | *CDKN2A* carriers vs. controls | NO |  |
| *LOC151484* | *CDKN2A* carriers vs. controls | NO |  |
| *LOC256880* | *CDKN2A* carriers vs. controls | NO |  |
| *LOC388282* | All melanoma patients vs. controls | NO |  |
| *LOC401557* | *CDKN2A* carriers vs. controls | NO |  |
| *LOC440028* | *CDKN2A* carriers vs. controls | NO |  |
| *LRIG2* | *CDKN2A* carriers vs. controls | YES | 17, 18 |
| *LSM5* | *CDKN2A* carriers vs. controls | YES | 18 |
| *LTN1* | *CDKN2A* carriers vs. controls | NO |  |
| *LYPD6* | *CDKN2A* WT vs. controls | YES | 17, 18 |
| *MCM6* | All melanoma patients vs. controls | NO |  |
| *MCM8* | All melanoma patients vs. controls | YES | 18 |
| *MCTP2* | *CDKN2A* carriers vs. controls | NO |  |
| *MDM1* | *CDKN2A* carriers vs. controls | YES | 18 |
| *ME1* | All melanoma patients vs. controls  *CDKN2A* carriers vs. controls | YES | 17, 18 |
| *MECOM* | All melanoma patients vs. controls | NO |  |
| *MELK* | All melanoma patients vs. controls | NO |  |
| *METTL14* | *CDKN2A* carriers vs. controls | NO |  |
| *MGC57346-CRHR1* | *CDKN2A* carriers vs. controls | NO |  |
| *MGP* | *CDKN2A* carriers vs. controls | NO |  |
| *MIPOL1* | *CDKN2A* carriers vs. controls | NO |  |
| *MIS18A* | All melanoma patients vs. controls | NO |  |
| *MITD1* | *CDKN2A* carriers vs. controls | NO |  |
| *MLLT4-AS1* | All melanoma patients vs. controls  *CDKN2A* carriers vs. controls | NO |  |
| *MMS22L* | *CDKN2A* carriers vs. controls | NO |  |
| *MOCS2* | All melanoma patients vs. controls  *CDKN2A* carriers vs. controls | NO |  |
| *MPC1* | *CDKN2A* carriers vs. controls | NO |  |
| *MPHOSPH9* | *CDKN2A* carriers vs. controls | NO |  |
| *MPRIP* | *CDKN2A* carriers vs. controls | NO |  |
| *MPV17L* | *CDKN2A* carriers vs. controls | NO |  |
| *MPZ* | *CDKN2A* carriers vs. controls | NO |  |
| *MROH7* | *CDKN2A* carriers vs. controls | NO |  |
| *MRPL1* | *CDKN2A* carriers vs. controls | NO |  |
| *MRPL19* | *CDKN2A* carriers vs. controls | NO |  |
| *MRPS14* | All melanoma patients vs. controls  *CDKN2A* carriers vs. controls | NO |  |
| *MSL3P1* | *CDKN2A* carriers vs. controls | NO |  |
| *MTERF2* | *CDKN2A* carriers vs. controls | NO |  |
| *MX2* | *CDKN2A* WT vs. controls | NO |  |
| *NCAPD2* | All melanoma patients vs. controls  *CDKN2A* carriers vs. controls | NO |  |
| *NCAPD3* | All melanoma patients vs. controls  *CDKN2A* carriers vs. controls | NO |  |
| *NCAPG2* | All melanoma patients vs. controls  *CDKN2A* carriers vs. controls | NO |  |
| *NEB* | *CDKN2A* carriers vs. controls | NO |  |
| *NEIL3* | All melanoma patients vs. controls  *CDKN2A* carriers vs. controls | NO |  |
| *NMI* | All melanoma patients vs. controls  *CDKN2A* carriers vs. controls  *CDKN2A* WT vs. controls | YES | 18 |
| *NUCKS1* | *CDKN2A* carriers vs. controls | NO |  |
| *NUDT16* | *CDKN2A* carriers vs. controls | NO |  |
| *NUF2* | All melanoma patients vs. controls | NO |  |
| *NUP107* | *CDKN2A* carriers vs. controls | YES | 17, 18 |
| *NUP205* | *CDKN2A* carriers vs. controls | YES | 17, 18 |
| *OAS1* | All melanoma patients vs. controls  *CDKN2A* carriers vs. controls  *CDKN2A* WT vs. controls | YES | 17, 18 |
| *OAS2* | All melanoma patients vs. controls  *CDKN2A* WT vs. controls | NO |  |
| *OAS3* | All melanoma patients vs. controls  *CDKN2A* WT vs. controls | NO |  |
| *OASL* | *CDKN2A* WT vs. controls | NO |  |
| *ODF3L1* | All melanoma patients vs. controls | NO |  |
| *OIP5-AS1* | *CDKN2A* carriers vs. controls | NO |  |
| *ORC6* | All melanoma patients vs. controls | NO |  |
| *ORMDL3* | *CDKN2A* carriers vs. controls | NO |  |
| *OSCAR* | *CDKN2A* carriers vs. controls | NO |  |
| *PARP12* | All melanoma patients vs. controls | NO |  |
| *PARP9* | All melanoma patients vs. controls  *CDKN2A* carriers vs. controls  *CDKN2A* WT vs. controls | NO |  |
| *PARPBP* | All melanoma patients vs. controls  *CDKN2A* carriers vs. controls | NO |  |
| *PCED1A* | *CDKN2A* carriers vs. controls | NO |  |
| *PCNA* | All melanoma patients vs. controls | YES | 18 |
| *PDCD1LG2* | All melanoma patients vs. controls | NO |  |
| *PHF11* | All melanoma patients vs. controls | YES | 17, 18 |
| *PHOSPHO1* | *CDKN2A* carriers vs. controls | YES | 18 |
| *PIGV* | *CDKN2A* carriers vs. controls | NO |  |
| *PKIB* | *CDKN2A* carriers vs. controls | YES | 17 |
| *PKP4* | *CDKN2A* carriers vs. controls | NO |  |
| *PLAC1* | All melanoma patients vs. controls | NO |  |
| *PLIN4* | All melanoma patients vs. controls  *CDKN2A* carriers vs. controls | NO |  |
| *PLSCR1* | All melanoma patients vs. controls  *CDKN2A* carriers vs. controls | NO |  |
| *POC1B* | *CDKN2A* carriers vs. controls | NO |  |
| *POLD3* | All melanoma patients vs. controls  *CDKN2A* carriers vs. controls | NO |  |
| *PPHLN1* | *CDKN2A* carriers vs. controls | YES | 18 |
| *PPP2R5E* | *CDKN2A* carriers vs. controls | NO |  |
| *PRDM10* | *CDKN2A* carriers vs. controls | NO |  |
| *PRKDC* | *CDKN2A* carriers vs. controls | NO |  |
| *PROP1* | *CDKN2A* carriers vs. controls | NO |  |
| *PRTFDC1* | All melanoma patients vs. controls  *CDKN2A* carriers vs. controls | NO |  |
| *PSMB8* | All melanoma patients vs. controls  *CDKN2A* WT vs. controls | YES | 18 |
| *PTPN13* | All melanoma patients vs. controls  *CDKN2A* carriers vs. controls | YES | 17, 18 |
| *RAB27A* | *CDKN2A* carriers vs. controls | NO |  |
| *RAD51* | All melanoma patients vs. controls | NO |  |
| *RAD51AP1* | *CDKN2A* carriers vs. controls | YES | 17, 18 |
| *RAD51D* | All melanoma patients vs. controls  *CDKN2A* carriers vs. controls | NO |  |
| *RAD54B* | *CDKN2A* carriers vs. controls | YES | 17, 18 |
| *RANBP1* | All melanoma patients vs. controls  *CDKN2A* carriers vs. controls | NO |  |
| *RBM26* | *CDKN2A* carriers vs. controls | NO |  |
| *RCC2* | *CDKN2A* carriers vs. controls | NO |  |
| *RDM1* | All melanoma patients vs. controls | NO |  |
| *REN* | *CDKN2A* carriers vs. controls | NO |  |
| *RFC5* | All melanoma patients vs. controls | NO |  |
| *RGS18* | *CDKN2A* carriers vs. controls | YES | 17, 18 |
| *RHPN1-AS1* | *CDKN2A* carriers vs. controls | NO |  |
| *RLN2* | All melanoma patients vs. controls  *CDKN2A* carriers vs. controls | NO |  |
| *RNA18S5* | *CDKN2A* carriers vs. controls | NO |  |
| *RNF180* | All melanoma patients vs. controls  *CDKN2A* carriers vs. controls | NO |  |
| *RNVU1-18* | All melanoma patients vs. controls | NO |  |
| *RP9P* | *CDKN2A* carriers vs. controls | NO |  |
| *RPGRIP1L* | *CDKN2A* carriers vs. controls | NO |  |
| *RSAD2* | *CDKN2A* WT vs. controls | NO |  |
| *RTKN2* | All melanoma patients vs. controls  *CDKN2A* carriers vs. controls | NO |  |
| *S100A10* | *CDKN2A* carriers vs. controls | NO |  |
| *SAMD13* | *CDKN2A* WT vs. controls | NO |  |
| *SASS6* | All melanoma patients vs. controls  *CDKN2A* carriers vs. controls | NO |  |
| *SCEL* | All melanoma patients vs. controls  *CDKN2A* carriers vs. controls | YES | 18 |
| *SCLT1* | All melanoma patients vs. controls  *CDKN2A* carriers vs. controls | NO |  |
| *SELM* | All melanoma patients vs. controls  *CDKN2A* carriers vs. controls | NO |  |
| *SEMA3D* | All melanoma patients vs. controls  *CDKN2A* carriers vs. controls  *CDKN2A* WT vs. controls | NO |  |
| *SEMA4D* | All melanoma patients vs. controls | YES | 18 |
| *SENP8* | *CDKN2A* carriers vs. controls | NO |  |
| *SFR1* | All melanoma patients vs. controls | NO |  |
| *SGOL2* | All melanoma patients vs. controls  *CDKN2A* carriers vs. controls | NO |  |
| *SHCBP1* | All melanoma patients vs. controls | NO |  |
| *SHOX* | *CDKN2A* carriers vs. controls | NO |  |
| *SIRT5* | *CDKN2A* carriers vs. controls | YES | 17, 18 |
| *SKA2* | *CDKN2A* carriers vs. controls | NO |  |
| *SLC10A7* | *CDKN2A* carriers vs. controls | NO |  |
| *SLC22A23* | *CDKN2A* carriers vs. controls | NO |  |
| *SLC25A3P1* | *CDKN2A* carriers vs. controls | NO |  |
| *SLC27A6* | All melanoma patients vs. controls | NO |  |
| *SLC28A1* | *CDKN2A* carriers vs. controls | NO |  |
| *SLC7A5P1* | *CDKN2A* carriers vs. controls | NO |  |
| *SLFN12* | All melanoma patients vs. controls | NO |  |
| *SMC2* | *CDKN2A* carriers vs. controls | YES | 17, 18 |
| *SNORA71A* | *CDKN2A* carriers vs. controls | NO |  |
| *SNX6* | All melanoma patients vs. controls  *CDKN2A* carriers vs. controls | YES | 18 |
| *SP110* | All melanoma patients vs. controls | NO |  |
| *SPATA17* | All melanoma patients vs. controls  *CDKN2A* carriers vs. controls | NO |  |
| *SRBD1* | *CDKN2A* carriers vs. controls | NO |  |
| *STAT1* | All melanoma patients vs. controls  *CDKN2A* WT vs. controls | YES | 18 |
| *STXBP5* | *CDKN2A* carriers vs. controls | YES | 17, 18 |
| *SUCLG2* | *CDKN2A* carriers vs. controls | NO |  |
| *TAF5L* | *CDKN2A* carriers vs. controls | NO |  |
| *TAP1* | All melanoma patients vs. controls  *CDKN2A* carriers vs. controls | YES | 17, 18 |
| *TBC1D19* | *CDKN2A* carriers vs. controls | YES | 18 |
| *TBC1D8B* | All melanoma patients vs. controls  *CDKN2A* carriers vs. controls | NO |  |
| *TCAIM* | *CDKN2A* carriers vs. controls | NO |  |
| *TCEAL4* | All melanoma patients vs. controls  *CDKN2A* carriers vs. controls | YES | 17, 18 |
| *TCF12* | *CDKN2A* carriers vs. controls | NO |  |
| *TCF19* | *CDKN2A* carriers vs. controls | YES | 17 |
| *TCF20* | *CDKN2A* carriers vs. controls | YES | 17, 18 |
| *TFCP2* | *CDKN2A* carriers vs. controls | NO |  |
| *TGDS* | *CDKN2A* carriers vs. controls | NO |  |
| *TGIF1* | *CDKN2A* carriers vs. controls | YES | 17, 18 |
| *TICRR* | All melanoma patients vs. controls  *CDKN2A* carriers vs. controls | NO |  |
| *TLE1* | *CDKN2A* carriers vs. controls | YES | 18 |
| *TMEM145* | All melanoma patients vs. controls | NO |  |
| *TMEM151A* | *CDKN2A* carriers vs. controls | NO |  |
| *TMEM194A* | *CDKN2A* carriers vs. controls | NO |  |
| *TMEM253* | All melanoma patients vs. controls | NO |  |
| *TMOD3* | *CDKN2A* carriers vs. controls | YES | 18 |
| *TMPO* | *CDKN2A* carriers vs. controls | NO |  |
| *TMPRSS11D* | All melanoma patients vs. controls  *CDKN2A* WT vs. controls | NO |  |
| *TNFRSF10D* | All melanoma patients vs. controls | NO |  |
| *TNNI3* | All melanoma patients vs. controls | NO |  |
| *TPX2* | All melanoma patients vs. controls | NO |  |
| *TRAIP* | All melanoma patients vs. controls | YES | 17 |
| *TRIM14* | All melanoma patients vs. controls  *CDKN2A* carriers vs. controls | NO |  |
| *TRIM21* | All melanoma patients vs. controls  *CDKN2A* WT vs. controls | NO |  |
| *TRIM36* | All melanoma patients vs. controls  *CDKN2A* WT vs. controls | NO |  |
| *TRIM5* | *CDKN2A* carriers vs. controls | NO |  |
| *TRIT1* | *CDKN2A* carriers vs. controls | YES | 17, 18 |
| *TRMT10A* | *CDKN2A* carriers vs. controls | NO |  |
| *TRRAP* | *CDKN2A* carriers vs. controls | NO |  |
| *TSGA10* | *CDKN2A* carriers vs. controls | NO |  |
| *TSKS* | *CDKN2A* carriers vs. controls | NO |  |
| *TTC13* | *CDKN2A* carriers vs. controls | YES | 17, 18 |
| *TTK* | All melanoma patients vs. controls  *CDKN2A* carriers vs. controls | YES | 18 |
| *TUB* | All melanoma patients vs. controls | NO |  |
| *TWF1* | All melanoma patients vs. controls  *CDKN2A* carriers vs. controls | NO |  |
| *UBE2L6* | All melanoma patients vs. controls | NO |  |
| *UBE2T* | All melanoma patients vs. controls  *CDKN2A* carriers vs. controls | YES | 17, 18 |
| *UEVLD* | *CDKN2A* carriers vs. controls | NO |  |
| *USHBP1* | *CDKN2A* carriers vs. controls | NO |  |
| *USP18* | All melanoma patients vs. controls  *CDKN2A* WT vs. controls | NO |  |
| *USP41* | *CDKN2A* WT vs. controls | NO |  |
| *USP8* | *CDKN2A* carriers vs. controls | NO |  |
| *VPREB3* | All melanoma patients vs. controls  *CDKN2A* carriers vs. controls | NO |  |
| *VRK1* | *CDKN2A* carriers vs. controls | NO |  |
| *WDHD1* | All melanoma patients vs. controls  *CDKN2A* carriers vs. controls | NO |  |
| *WDR86* | All melanoma patients vs. controls | NO |  |
| *XAF1* | All melanoma patients vs. controls | YES | 17, 18 |
| *XLOC_l2_001687* | *CDKN2A* carriers vs. controls | NO |  |
| *XRCC3* | All melanoma patients vs. controls | YES | 17, 18 |
| *YWHAZ* | *CDKN2A* carriers vs. controls | NO |  |
| *ZBED8* | *CDKN2A* carriers vs. controls | NO |  |
| *ZC2HC1C* | *CDKN2A* carriers vs. controls | NO |  |
| *ZCWPW2* | *CDKN2A* carriers vs. controls | NO |  |
| *ZFP69* | All melanoma patients vs. controls | NO |  |
| *ZNF252P* | *CDKN2A* carriers vs. controls | NO |  |
| *ZNF333* | *CDKN2A* carriers vs. controls | YES | 17, 18 |
| *ZNF480* | *CDKN2A* carriers vs. controls | YES | 17, 18 |
| *ZNF518A* | *CDKN2A* carriers vs. controls | YES | 17, 18 |
| *ZSCAN20* | *CDKN2A* carriers vs. controls | NO |  |
| *ZWILCH* | All melanoma patients vs. controls | NO |  |
| *ZWINT* | All melanoma patients vs. controls | NO |  |

All statistically significant genes in our study are listed in the table.

Blue: Upregulated genes; Red: Downregulated genes

**Table S8. Open Targets platform association score for each significant DEG in breast cancer and melanoma phenotype**

| **Gene** | **Breast (text mining)** | **Breast (Genetic association)** | **Melanoma (text mining)** | **Melanoma (Genetic association)** |
| --- | --- | --- | --- | --- |
| *AATK* | 0.2339267 | 0.02844225 | 0.16602083 | 0.02018584 |
| *ACLY* | 0.31108784 | 0.03782398 | 0.21881287 | 0.02660462 |
| *ACTR3* | 0.07599135 | 0.0092395 | 0.07176961 | 0.00872619 |
| *ACTR6* | - | - | - | - |
| *ADAMTSL3* | - | 0.15388169 | 0.03039654 | 0.0036958 |
| *AIM2* | 0.28841554 | 0.03506734 | 0.65822526 | 0.12118748 |
| *ALMS1P* | - | - | - | - |
| *AMDHD1* | 0.06079308 | 0.0073916 | - | 0.10025575 |
| *ANP32E* | 0.61064171 | 0.07424558 | - | - |
| *APC* | 0.91523739 | 0.52495862 | 0.26447586 | 0.44454824 |
| *APITD1* | - | - | - | - |
| *ARPC2* | 0.68875172 | 0.20238341 | 0.02127758 | 0.00258706 |
| *ASB9* | 0.05319394 | 0.00646765 | - | - |
| *ASPG* | 0.06383273 | 0.00776118 | 0.01215862 | 0.00147832 |
| *ASPM* | 0.50178359 | 0.06100994 | 0.08519145 | 0.0103581 |
| *ATAD5* | 0.03647585 | 0.29851911 | - | - |
| *ATE1-AS1* | - | - | - | - |
| *ATG10* | 0.12071638 | 0.38981512 | 0.04863446 | 0.00591328 |
| *AURKA* | 0.87897123 | 0.31880242 | 0.71844458 | 0.08735292 |
| *AURKB* | 0.58978794 | 0.29118895 | 0.70739418 | 0.08600934 |
| *BCAR1* | 0.86131145 | 0.2899663 | 0.14651307 | 0.16679229 |
| *BCCIP* | 0.20163038 | 0.02451546 | - | - |
| *BCKDHB* | - | 0.15654888 | - | - |
| *BEND6* | - | - | - | - |
| *BET1L* | - | 0.10377897 | - | - |
| *BEX5* | - | - | - | - |
| *BLM* | 0.46427568 | 0.54993108 | 0.07204487 | 0.39101872 |
| *BMP2K* | 0.14894305 | 0.01810941 | - | - |
| *BORA* | 0.61840072 | 0.24731806 | - | - |
| *BRCA1* | 0.99635884 | 0.91014954 | 0.79434456 | 0.51853538 |
| *BRCA2* | 0.97995747 | 0.90395274 | 0.36301284 | 0.57096306 |
| *BRIP1* | 0.8714748 | 0.8025969 | 0.15580422 | 0.39356471 |
| *BTBD8* | - | - | - | - |
| *BTG4* | 0.01823792 | 0.00221748 | 0.01215862 | 0.00147832 |
| *C14orf180* | - | - | - | - |
| *C14orf80* | - | - | - | - |
| *C16orf59* | - | - | - | - |
| *C17orf85* | - | - | - | - |
| *C18orf54* | - | - | - | - |
| *C1orf112* | - | - | - | - |
| *C20orf62* | - | - | - | - |
| *C2CD5* | 0.06535256 | 0.00794597 | - | - |
| *C5orf15* | - | - | - | - |
| *C6orf211* | - | - | - | - |
| *C7orf61* | - | - | - | - |
| *C8orf48* | 0.01215862 | 0.00147832 | - | - |
| *CACTIN* | - | - | - | - |
| *CASC1* | - | - | - | - |
| *CASC5* | - | - | - | - |
| *CASD1* | 0.70186455 | 0.12596551 | 0.01215862 | 0.00147832 |
| *CASP8* | 0.81318828 | 0.58103417 | 0.19722018 | 0.45318063 |
| *CASS4* | - | - | - | - |
| *CATSPER3* | - | 0.16060342 | - | 0.24972016 |
| *CBL* | 0.58420543 | 0.50248213 | 0.65535402 | 0.5990948 |
| *CCDC153* | - | - | - | - |
| *CCDC50* | - | - | - | - |
| *CCDC88C* | - | 0.41990862 | 0.45442827 | 0.05525219 |
| *CDC23* | 0.01519827 | 0.0018479 | - | - |
| *CDC25C* | 0.20784423 | 0.28580848 | 0.17131828 | 0.02082993 |
| *CDNF* | 0.03039654 | 0.0036958 | - | - |
| *CENPA* | 0.23675613 | 0.02878627 | - | - |
| *CENPE* | 0.26699007 | 0.18914928 | 0.06383273 | 0.00776118 |
| *CENPI* | 0.08769739 | 0.01066279 | - | - |
| *CENPK* | 0.05237155 | 0.00636766 | 0.15704879 | 0.01909496 |
| *CENPQ* | - | - | - | - |
| *CEP128* | - | - | - | - |
| *CEP295* | - | - | - | - |
| *CFAP46* | - | - | - | - |
| *CFDP1* | 0.1615279 | 0.01963956 | 0.21689648 | 0.02637161 |
| *CHIA* | - | - | - | - |
| *CHM* | 0.05471377 | 0.24902548 | - | 0.13376532 |
| *CHRM3* | 0.04002211 | 0.26492993 | 0.03039654 | 0.20128758 |
| *CITED2* | 0.36408793 | 0.21553283 | 0.05471377 | 0.00665244 |
| *CKAP2* | 0.45894553 | 0.05580143 | - | - |
| *CLSPN* | 0.18615854 | 0.28728663 | 0.16110166 | 0.01958773 |
| *CLTC* | 0.13727719 | 0.57234702 | 0.50306274 | 0.45337182 |
| *CLUAP1* | - | - | - | - |
| *CMPK2* | 0.148301 | 0.01803135 | 0.06383273 | 0.00776118 |
| *CMTR2* | - | - | - | - |
| *CNOT3* | - | 0.45785821 | - | 0.4696744 |
| *COMMD10* | - | - | - | - |
| *COMMD8* | - | - | - | - |
| *CPEB3* | 0.01215862 | 0.27672598 | 0.61840072 | 0.27609155 |
| *CPNE8* | 0.05901995 | 0.00717601 | - | - |
| *CPPED1* | 0.01215862 | 0.00147832 | - | - |
| *CREM* | 0.03647585 | 0.00443496 | 0.13374478 | 0.01626151 |
| *CSE1L* | 0.4210731 | 0.05119666 | 0.38376691 | 0.04666075 |
| *CSF2RA* | 0.16751871 | 0.42421479 | - | 0.56532768 |
| *CTSB* | 0.68385096 | 0.26797267 | 0.81122119 | 0.09863327 |
| *CYP2J2* | 0.08436887 | 0.01025809 | 0.06079308 | 0.23283531 |
| *DARS2* | - | - | - | - |
| *DDIAS* | 0.09422927 | 0.01145698 | - | - |
| *DDX60* | 0.13070512 | 0.01589193 | 0.10942754 | 0.01330487 |
| *DHTKD1* | - | - | - | - |
| *DHX30* | 0.04255516 | 0.00517412 | - | - |
| *DMC1* | 0.01823792 | 0.22490016 | 0.01215862 | 0.00147832 |
| *DNAAF3* | 0.02338845 | 0.00284371 | - | - |
| *DSCAM* | 0.26066835 | 0.18921714 | 0.10334824 | 0.18793136 |
| *DSCC1* | 0.13678443 | 0.01663109 | - | - |
| *DUSP19* | - | - | - | - |
| *DUSP26* | 0.0729517 | 0.00886992 | - | - |
| *DZIP3* | 0.01215862 | 0.00147832 | - | - |
| *E2F8* | 0.38273764 | 0.0465356 | 0.04407498 | 0.00535891 |
| *ECT2* | 0.72208027 | 0.20319241 | 0.03647585 | 0.00443496 |
| *EDEM2* | - | - | - | - |
| *EFCAB11* | - | - | - | 0.16290413 |
| *EHHADH* | - | - | - | - |
| *EIF2AK2* | 0.81876555 | 0.09955056 | 0.17377199 | 0.02112827 |
| *EIF3F* | 0.03039654 | 0.0036958 | 0.37420164 | 0.04549774 |
| *EMP1* | 0.21590952 | 0.02625161 | 0.03343619 | 0.00406538 |
| *ENTPD1-AS1* | - | - | - | - |
| *EPS8L3* | - | - | - | - |
| *ERI2* | 0.88293649 | 0.10735286 | 0.72173222 | 0.08775265 |
| *ESCO2* | 0.01519827 | 0.0018479 | 0.01519827 | 0.0018479 |
| *EXO1* | 0.69629595 | 0.48974129 | 0.15806201 | 0.01921815 |
| *EXOC4* | 0.01215862 | 0.00147832 | - | - |
| *EXTL2* | - | - | - | 0.00098461 |
| *FAM107B* | 0.02127758 | 0.00258706 | - | - |
| *FAM111B* | 0.09118962 | 0.0110874 | - | - |
| *FAM122B* | - | - | - | - |
| *FAM129A* | - | - | - | - |
| *FAM161A* | - | - | - | - |
| *FAM178A* | - | - | - | - |
| *FAM186B* | - | - | - | - |
| *FAM208A* | - | - | - | - |
| *FAM20C* | 0.08511031 | 0.01034824 | - | - |
| *FAM72D* | - | - | - | - |
| *FANCA* | 0.51727509 | 0.47383252 | 0.28616147 | 0.45967232 |
| *FANCB* | 0.09694876 | 0.21559669 | 0.04103533 | 0.00498933 |
| *FANCD2* | 0.49401409 | 0.5910174 | 0.43764337 | 0.42611908 |
| *FANCI* | 0.193672 | 0.28649457 | - | - |
| *FERMT1* | 0.14157695 | 0.0172138 | 0.03039654 | 0.0036958 |
| *FGFR1OP* | - | - | - | - |
| *FKTN* | 0.01215862 | 0.00147832 | - | - |
| *FMO5* | 0.04255516 | 0.00517412 | - | - |
| *FOLH1* | 0.46641276 | 0.05670934 | 0.11060507 | 0.01344805 |
| *FOLH1B* | - | - | - | - |
| *FTCDNL1* | - | - | - | - |
| *G2E3* | - | - | - | - |
| *GCM1* | 0.02127758 | 0.00258706 | - | - |
| *GDF7* | - | - | - | - |
| *GEN1* | 0.19602971 | 0.2575056 | - | - |
| *GINS2* | 0.252325 | 0.03067923 | 0.06018177 | 0.00731727 |
| *GLCE* | 0.6736453 | 0.08190595 | - | - |
| *GLYCTK* | - | - | - | - |
| *GNG10* | - | 0.27822323 | 0.03706689 | 0.00450682 |
| *GNL1* | 0.01823792 | 0.00221748 | - | - |
| *GP6* | 0.071263 | 0.24869754 | 0.01215862 | 0.00147832 |
| *GPC6* | 0.05302508 | 0.16032924 | 0.01654923 | 0.24203581 |
| *GPR161* | 0.09704164 | 0.01179892 | - | - |
| *GPR3* | 0.14130169 | 0.01718033 | - | - |
| *GSAP* | - | - | - | - |
| *GSTCD* | - | 0.00193422 | - | - |
| *GTF2H3* | 0.07860628 | 0.2516194 | 0.08561692 | 0.01040983 |
| *GZMM* | 0.14897141 | 0.01811286 | - | 0.10132564 |
| *HAPLN2* | - | - | - | - |
| *HCCAT5* | - | - | - | - |
| *HCG26* | - | - | - | - |
| *HCP5* | 0.0916122 | 0.01113878 | 0.16458544 | 0.02001131 |
| *HEMK1* | - | - | - | - |
| *HEPH* | 0.75265321 | 0.09151221 | 0.07648959 | 0.00930008 |
| *HERC5* | 0.06839221 | 0.00831555 | 0.03039654 | 0.0036958 |
| *HERC6* | - | 0.06569264 | - | - |
| *HLA-F* | 0.16041257 | 0.01950395 | 0.09102075 | 0.01106686 |
| *HLA-G* | 0.82298076 | 0.10006307 | 0.44291683 | 0.05385256 |
| *HLA-J* | 0.14590339 | 0.01773983 | - | - |
| *HMGN5* | 0.16726108 | 0.02033663 | - | - |
| *HN1* | - | - | - | - |
| *HOXA6* | 0.03630698 | 0.00441443 | 0.03039654 | 0.0036958 |
| *HOXA7* | 0.1133925 | 0.01378696 | 0.03039654 | 0.0036958 |
| *HOXA9* | 0.57034736 | 0.39589928 | 0.47030867 | 0.38130908 |
| *HSH2D* | 0.02127758 | 0.00258706 | - | - |
| *HSPG2* | 0.20270503 | 0.27593647 | 0.40381526 | 0.25380736 |
| *ICA1L* | - | - | - | - |
| *IFI44* | 0.1299391 | 0.0157988 | 0.12571345 | 0.01528502 |
| *IFI44L* | 0.0729517 | 0.00886992 | 0.03799567 | 0.00461975 |
| *IFI6* | 0.65722628 | 0.07990962 | 0.61688089 | 0.07500418 |
| *IFIH1* | 0.09874654 | 0.2489546 | 0.16409063 | 0.01995115 |
| *IFIT1* | 0.19010216 | 0.02311379 | 0.10707519 | 0.01301886 |
| *IFITM4P* | - | - | - | - |
| *IFT88* | 0.06687239 | 0.00813076 | - | - |
| *IGF2BP3* | 0.30390205 | 0.03695028 | 0.70875875 | 0.08617525 |
| *IL15RA* | 0.45493488 | 0.05531378 | 0.08167044 | 0.00992999 |
| *IQCH* | - | - | - | - |
| *IRF7* | 0.13652062 | 0.18518346 | 0.17722027 | 0.02154753 |
| *IRF9* | 0.5727391 | 0.06963715 | 0.0879811 | 0.01069728 |
| *IRX1* | - | - | - | - |
| *ISG15* | 0.83153442 | 0.10110308 | 0.09228727 | 0.01122086 |
| *JAK2* | 0.73250886 | 0.51809765 | 0.77627282 | 0.45197091 |
| *JMJD6* | 0.78095865 | 0.24794337 | 0.69584509 | 0.08460513 |
| *JRKL* | - | - | - | - |
| *KBTBD3* | - | - | - | - |
| *KCNQ4* | 0.06079308 | 0.02444963 | - | - |
| *KDM3B* | - | 0.22420493 | - | - |
| *KDM4C* | 0.74013285 | 0.24670241 | 0.01215862 | 0.00147832 |
| *KIAA0895* | - | - | - | - |
| *KIAA1107* | - | - | - | - |
| *KIAA1841* | - | - | - | - |
| *KIF14* | 0.41500412 | 0.11484503 | - | - |
| *KIF20B* | 0.04711464 | 0.18246582 | - | - |
| *KIF21A* | 0.01823792 | 0.18158807 | - | - |
| *KIF24* | 0.21121373 | 0.02568067 | - | - |
| *KLHL12* | - | - | - | - |
| *KLRG2* | - | - | - | - |
| *KRT31* | - | 0.17976301 | 0.26951599 | 0.03276941 |
| *LAMP2* | 0.25458315 | 0.25306589 | 0.20938477 | 0.02545829 |
| *LAMP3* | 0.27394413 | 0.03330781 | 0.13287413 | 0.01615566 |
| *LAP3* | 0.16636134 | 0.02022724 | 0.07197449 | 0.0087511 |
| *LARP1B* | - | - | - | - |
| *LIMK1* | 0.68577288 | 0.20229287 | 0.08050861 | 0.00978873 |
| *LINC00106* | - | - | - | - |
| *LINC01118* | - | - | - | - |
| *LINC01133* | 0.34652055 | 0.0421321 | - | - |
| *LINC01341* | - | - | - | - |
| *LINC01478* | - | - | - | - |
| *LINC01510* | - | - | - | - |
| *LINGO2* | - | - | - | - |
| *LIPA* | - | - | 0.33132228 | 0.19486098 |
| *LMF1* | - | - | - | 0.11125239 |
| *LMX1B* | - | 0.31196136 | - | - |
| *lnc-AGPAT6-1* | - | - | - | - |
| *lnc-AKR1C2-4* | - | - | - | - |
| *lnc-CARHSP1-1* | - | - | - | - |
| *lnc-GLIPR1-3* | - | - | - | - |
| *lnc-INTS9-1* | - | - | - | - |
| *lnc-ITGA2-1* | - | - | - | - |
| *lnc-PPP3CA-1* | - | - | - | - |
| *lnc-SNURF-3* | - | - | - | - |
| *LOC100128882* | - | - | - | - |
| *LOC100132057* | - | - | - | - |
| *LOC100132368* | - | - | - | - |
| *LOC100133286* | - | - | - | - |
| *LOC100288842* | - | - | - | - |
| *LOC101059906* | - | - | - | - |
| *LOC101927910* | - | - | - | - |
| *LOC151484* | - | - | - | - |
| *LOC256880* | - | - | - | - |
| *LOC388282* | - | - | - | - |
| *LOC401557* | - | - | - | - |
| *LOC440028* | - | - | - | - |
| *LRIG2* | 0.03647585 | 0.00443496 | 0.11533798 | 0.0140235 |
| *LSM5* | - | - | - | - |
| *LTN1* | - | - | - | - |
| *LYPD6* | - | - | - | - |
| *MCM6* | 0.15842022 | 0.22994333 | 0.08194401 | 0.00996326 |
| *MCM8* | 0.07457791 | 0.32333808 | 0.29591866 | 0.03597961 |
| *MCTP2* | - | - | - | - |
| *MDM1* | 0.12158616 | 0.01478319 | - | - |
| *ME1* | 0.66017625 | 0.08026829 | 0.02127758 | 0.00258706 |
| *MECOM* | 0.70862707 | 0.6043519 | 0.26293007 | 0.53654453 |
| *MELK* | 0.898748 | 0.10927532 | 0.48922056 | 0.05948245 |
| *METTL14* | 0.74855198 | 0.09101356 | 0.13374478 | 0.01626151 |
| *MGC57346-CRHR1* | - | - | - | - |
| *MGP* | 0.29974862 | 0.03644528 | 0.03039654 | 0.0036958 |
| *MIPOL1* | 0.01823792 | 0.00221748 | - | - |
| *MIS18A* | - | - | - | - |
| *MITD1* | 0.01215862 | 0.00147832 | - | - |
| *MLLT4-AS1* | - | - | - | - |
| *MMS22L* | 0.03039654 | 0.0036958 | - | - |
| *MOCS2* | - | - | - | - |
| *MPC1* | 0.02127758 | 0.00258706 | - | - |
| *MPHOSPH9* | - | - | - | - |
| *MPRIP* | 0.17317254 | 0.18563206 | - | - |
| *MPV17L* | 0.03039654 | 0.0036958 | - | - |
| *MPZ* | - | - | 0.06079308 | 0.0073916 |
| *MROH7* | - | - | - | - |
| *MRPL1* | - | - | - | - |
| *MRPL19* | 0.04559481 | 0.0055437 | 0.04863446 | 0.00591328 |
| *MRPS14* | - | - | - | - |
| *MSL3P1* | - | - | - | - |
| *MTERF2* | - | - | - | - |
| *MX2* | - | - | 0.67246761 | 0.47202849 |
| *NCAPD2* | 0.09118962 | 0.0110874 | 0.03039654 | 0.0036958 |
| *NCAPD3* | - | 0.17937016 | - | - |
| *NCAPG2* | 0.21352441 | 0.02596161 | 0.04086646 | 0.0049688 |
| *NEB* | 0.04686133 | 0.22668904 | - | - |
| *NEIL3* | 0.079031 | 0.00960908 | - | - |
| *NMI* | 0.70530704 | 0.08575557 | 0.02262854 | 0.00275132 |
| *NUCKS1* | 0.31522137 | 0.03832656 | - | - |
| *NUDT16* | - | - | - | - |
| *NUF2* | 0.3200704 | 0.03891613 | 0.15181383 | 0.01845846 |
| *NUP107* | - | 0.24540527 | - | - |
| *NUP205* | - | 0.24540527 | - | - |
| *OAS1* | 0.33729689 | 0.04101063 | 0.03039654 | 0.0036958 |
| *OAS2* | 0.22158302 | 0.02694143 | 0.07041865 | 0.00856193 |
| *OAS3* | 0.36475848 | 0.04434958 | 0.09118962 | 0.0110874 |
| *OASL* | 0.31764384 | 0.04472135 | 0.03039654 | 0.0036958 |
| *ODF3L1* | - | - | - | - |
| *OIP5-AS1* | 0.44675652 | 0.05431941 | 0.13805095 | 0.01678509 |
| *ORC6* | 0.04137307 | 0.00503039 | - | - |
| *ORMDL3* | 0.10816102 | 0.01315088 | - | - |
| *OSCAR* | - | - | 0.01215862 | 0.00147832 |
| *PARP12* | 0.04407498 | 0.00535891 | 0.03039654 | 0.0036958 |
| *PARP9* | 0.17933959 | 0.02180521 | - | - |
| *PARPBP* | 0.64097859 | 0.07793413 | - | - |
| *PCED1A* | - | - | - | - |
| *PCNA* | 0.41336368 | 0.0502593 | 0.26770814 | 0.0325496 |
| *PDCD1LG2* | 0.16004584 | 0.51657895 | 0.4222567 | 0.44689046 |
| *PHF11* | 0.09118962 | 0.0110874 | - | - |
| *PHOSPHO1* | - | - | - | - |
| *PIGV* | - | - | - | - |
| *PKIB* | 0.16829044 | 0.02046179 | - | - |
| *PKP4* | 0.06839221 | 0.22670545 | - | - |
| *PLAC1* | 0.73771406 | 0.08969582 | 0.12513242 | 0.01521437 |
| *PLIN4* | 0.06518369 | 0.00792543 | - | - |
| *PLSCR1* | 0.70097798 | 0.08522922 | - | - |
| *POC1B* | - | - | - | - |
| *POLD3* | 0.76723983 | 0.66367255 | 0.01215862 | 0.3512079 |
| *PPHLN1* | 0.02387479 | 0.00290284 | 0.1015751 | 0.01235013 |
| *PPP2R5E* | 0.01823792 | 0.29492268 | - | 0.24842661 |
| *PRDM10* | 0.06835 | 0.03972046 | 0.04711464 | 0.00572849 |
| *PRKDC* | 0.70114193 | 0.24744912 | 0.18437263 | 0.02241716 |
| *PROP1* | - | - | - | - |
| *PRTFDC1* | - | - | - | - |
| *PSMB8* | 0.17072723 | 0.30161223 | 0.39085784 | 0.0475229 |
| *PTPN13* | 0.57074228 | 0.62156638 | 0.04116198 | 0.39585467 |
| *RAB27A* | 0.38624065 | 0.04696152 | 0.26515532 | 0.03223922 |
| *RAD51* | 0.86790467 | 0.5821455 | 0.78767601 | 0.0957705 |
| *RAD51AP1* | 0.07753313 | 0.26052315 | 0.16093279 | 0.0195672 |
| *RAD51D* | 0.40664113 | 0.7263723 | - | - |
| *RAD54B* | 0.09793079 | 0.01190703 | 0.01215862 | 0.00147832 |
| *RANBP1* | 0.01823792 | 0.00221748 | 0.01654923 | 0.00201216 |
| *RBM26* | - | - | - | - |
| *RCC2* | 0.23416123 | 0.02847076 | 0.11229833 | 0.01365392 |
| *RDM1* | 0.6079308 | 0.07391597 | - | - |
| *REN* | 0.16391524 | 0.01992982 | 0.03799567 | 0.13492026 |
| *RFC5* | 0.03343619 | 0.28452261 | - | - |
| *RGS18* | - | - | - | - |
| *RHPN1-AS1* | - | - | - | - |
| *RLN2* | 0.16243812 | 0.17452268 | 0.05781111 | 0.18654719 |
| *RNA18S5* | - | - | - | - |
| *RNF180* | - | - | - | - |
| *RNVU1-18* | - | - | - | - |
| *RP9P* | - | - | - | - |
| *RPGRIP1L* | 0.03039654 | 0.18250412 | - | - |
| *RSAD2* | 0.06079308 | 0.0073916 | 0.01823792 | 0.00221748 |
| *RTKN2* | - | - | - | - |
| *S100A10* | 0.79103809 | 0.09617928 | 0.15350253 | 0.01866378 |
| *SAMD13* | - | - | - | - |
| *SASS6* | - | - | - | - |
| *SCEL* | - | - | - | - |
| *SCLT1* | - | - | - | - |
| *SELM* | - | - | - | - |
| *SEMA3D* | 0.03039654 | 0.0036958 | 0.03039654 | 0.0036958 |
| *SEMA4D* | 0.6747666 | 0.08204228 | 0.06427686 | 0.10804912 |
| *SENP8* | - | 0.18076 | - | - |
| *SFR1* | 0.41339294 | 0.05026286 | - | - |
| *SGOL2* | - | - | - | - |
| *SHCBP1* | 0.22445582 | 0.02729072 | 0.02431723 | 0.00295664 |
| *SHOX* | - | - | - | - |
| *SIRT5* | 0.20888073 | 0.02539701 | 0.15841157 | 0.01926065 |
| *SKA2* | 0.77236101 | 0.09390841 | - | - |
| *SLC10A7* | 0.2065276 | 0.0251109 | - | - |
| *SLC22A23* | 0.03647585 | 0.00443496 | - | - |
| *SLC25A3P1* | - | - | - | - |
| *SLC27A6* | 0.01823792 | 0.00221748 | - | 0.14680998 |
| *SLC28A1* | 0.08689526 | 0.01056526 | - | 0.12116744 |
| *SLC7A5P1* | - | - | - | - |
| *SLFN12* | 0.11854651 | 0.01441361 | 0.04407498 | 0.00535891 |
| *SMC2* | 0.25212241 | 0.25286322 | 0.15552896 | 0.01891017 |
| *SNORA71A* | 0.03039654 | 0.0036958 | - | - |
| *SNX6* | - | - | - | - |
| *SP110* | - | - | - | - |
| *SPATA17* | - | - | - | - |
| *SRBD1* | - | - | - | - |
| *STAT1* | 0.9404428 | 0.1423294 | 0.73511115 | 0.08937934 |
| *STXBP5* | 0.0227974 | 0.00277185 | - | - |
| *SUCLG2* | - | - | - | - |
| *TAF5L* | 0.15046287 | 0.22877848 | - | - |
| *TAP1* | 0.27563527 | 0.03351343 | 0.80044449 | 0.09732297 |
| *TBC1D19* | - | - | 0.0562336 | 0.00683723 |
| *TBC1D8B* | - | - | 0.01215862 | 0.00147832 |
| *TCAIM* | - | - | - | - |
| *TCEAL4* | - | - | - | - |
| *TCF12* | 0.0346301 | 0.28111624 | 0.06683017 | 0.25932571 |
| *TCF19* | 0.03039654 | 0.0036958 | 0.42673365 | 0.05188491 |
| *TCF20* | 0.04256698 | 0.00517556 | - | - |
| *TFCP2* | 0.14295482 | 0.01738133 | 0.6226765 | 0.07570884 |
| *TGDS* | - | - | - | - |
| *TGIF1* | 0.35292929 | 0.04291132 | - | - |
| *TICRR* | 0.21708196 | 0.02639416 | - | - |
| *TLE1* | 0.36393242 | 0.21464683 | 0.13813539 | 0.01679535 |
| *TMEM145* | - | - | - | - |
| *TMEM151A* | - | - | - | - |
| *TMEM194A* | - | - | - | - |
| *TMEM253* | - | - | - | - |
| *TMOD3* | 0.02431723 | 0.22600378 | - | - |
| *TMPO* | 0.400282 | 0.04866875 | 0.2122641 | 0.02580838 |
| *TMPRSS11D* | 0.46251145 | 0.05623499 | 0.65447769 | 0.07957543 |
| *TNFRSF10D* | 0.09113313 | 0.26161522 | 0.29483258 | 0.19375182 |
| *TNNI3* | 0.01215862 | 0.25515863 | - | - |
| *TPX2* | 0.35187317 | 0.27580157 | 0.14197717 | 0.01726246 |
| *TRAIP* | 0.3028897 | 0.0368272 | 0.06670352 | 0.00811022 |
| *TRIM14* | 0.18904959 | 0.02298581 | - | - |
| *TRIM21* | 0.37147083 | 0.11203049 | 0.48712259 | 0.05922736 |
| *TRIM36* | - | - | - | - |
| *TRIM5* | 0.01215862 | 0.00147832 | - | - |
| *TRIT1* | 0.03039654 | 0.0036958 | 0.01823792 | 0.00221748 |
| *TRMT10A* | - | - | - | - |
| *TRRAP* | 0.81440846 | 0.58767476 | 0.42832582 | 0.56532156 |
| *TSGA10* | 0.58895491 | 0.07160877 | 0.01823792 | 0.00221748 |
| *TSKS* | - | - | - | - |
| *TTC13* | - | - | - | - |
| *TTK* | 0.65516677 | 0.07965921 | 0.14080277 | 0.01711967 |
| *TUB* | 0.03039654 | 0.0036958 | - | - |
| *TWF1* | 0.36271843 | 0.04410154 | - | - |
| *UBE2L6* | 0.0723826 | 0.18183902 | - | - |
| *UBE2T* | 0.34728942 | 0.19019524 | - | - |
| *UEVLD* | 0.03039654 | 0.0036958 | - | - |
| *USHBP1* | - | 0.14967538 | - | - |
| *USP18* | 0.24796484 | 0.03014909 | 0.18888072 | 0.02296528 |
| *USP41* | 0.03495602 | 0.00425017 | - | - |
| *USP8* | 0.09858949 | 0.56717278 | 0.04103533 | 0.39007614 |
| *VPREB3* | - | - | - | - |
| *VRK1* | 0.80580089 | 0.09797424 | 0.03343619 | 0.00406538 |
| *WDHD1* | 0.46729982 | 0.05681719 | 0.07648959 | 0.00930008 |
| *WDR86* | 0.16414132 | 0.01995731 | - | - |
| *XAF1* | 0.04542594 | 0.00552317 | 0.15748944 | 0.01914854 |
| *XLOC_l2_001687* | - | - | - | - |
| *XRCC3* | 0.81494852 | 0.51918191 | 0.27189657 | 0.48457321 |
| *YWHAZ* | 0.21680197 | 0.28915857 | 0.04610142 | 0.00560529 |
| *ZBED8* | - | - | - | - |
| *ZC2HC1C* | - | - | - | - |
| *ZCWPW2* | - | - | - | - |
| *ZFP69* | - | - | - | - |
| *ZNF252P* | - | - | - | - |
| *ZNF333* | - | - | - | - |
| *ZNF480* | - | - | - | - |
| *ZNF518A* | - | - | - | - |
| *ZSCAN20* | - | - | - | - |
| *ZWILCH* | 0.01215862 | 0.00147832 | - | - |
| *ZWINT* | 0.29744365 | 0.03616503 | 0.10486806 | 0.0127505 |

Blue: Upregulated genes; Red: Downregulated genes

Association score is between 0 and 1, the higher the score, the stronger the association.

## Supplementary Figures

**Supplementary Figure 1.** **Principal component analyses and heatmap of a comparison of the significant differentially expressed genes according to comparisons**. MM: melanoma, C: control, +: *CDKN2A* mutant patients, -: *CDKN2A* wild-type patients. The figure shows two principal component plots of the first component (x-axis) vs. the second component (y-axis), left side, and the third component (x-axis) vs. the second component (y-axis), right side. Below the plots, a heatmap of the significant gene signature for each comparison is shown. No significant genes were detected in the *CDKN2A* mutant vs. wild-type melanoma patients.


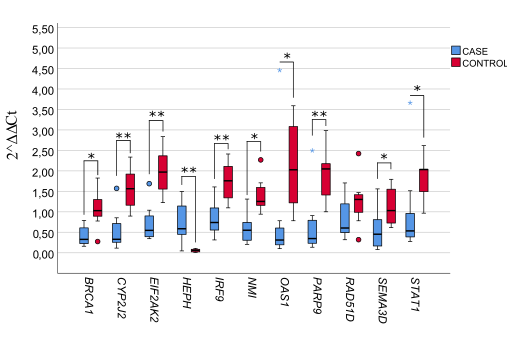
**Supplementary Figure 2.** **qPCR results boxplot**. The figure shows the 2^ΔΔCt values of cases and controls for each gene. Mann-Whitney U test was performed. * p < 0.05; ** p <0,001


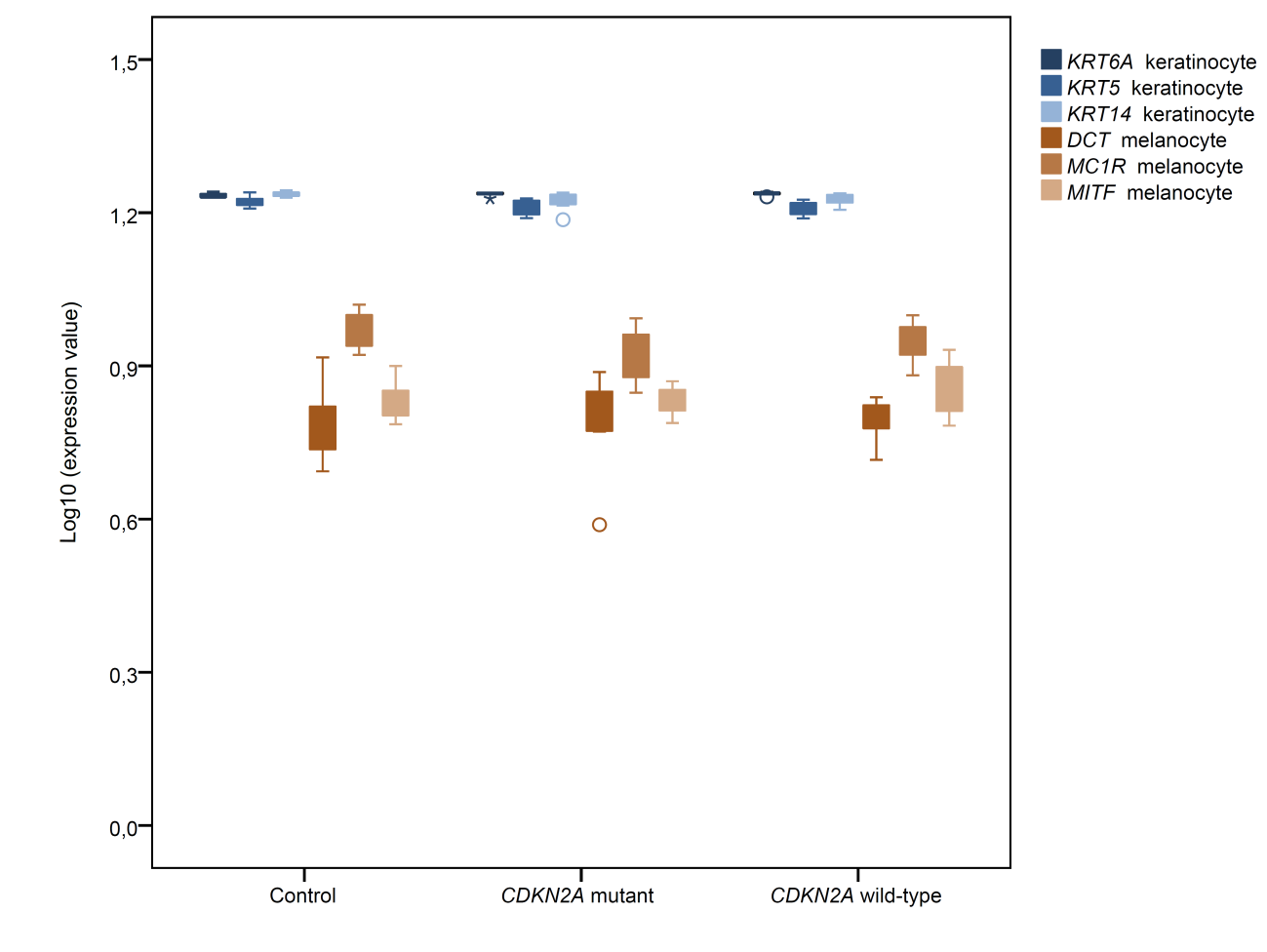


**Supplementary Figure 3. Log10 gene expression of keratinocyte and melanocyte specific genes among study groups.** Blue: keratinocyte-specific gene expression. Brown: melanocyte-specific gene expression. Expression values obtained after data Normalization in Babelomics were log10-transformed for further normalization prior to plotting. Anova test was carried out to assess differences between groups. No statistically significant differences were observed between controls, *CDKN2A* mutant or *CDKN2A* wild-type patients. P-values after FDR correction for multiple test comparisons according to each gene were: *KRT6A* 0.321, *KRT5* 0.252, *KRT14* 0.252, *CCDT* 0.953*, MC1R* 0.282, *MITF* 0.419.

# Supplementary data

## Analyses by sex

In order to assess whether sex could have an influence on the main results we performed analyses by sex. We used the same bioinformatic tools and statistical tests described in the manuscript.

### Female comparison

We used information from melanocyte-keratinocyte co-cultures derived from healthy skin from female melanoma patients (N=9) and female controls (N=2).

Whole transcriptome expression data analysis comparing female cases vs. female controls showed three differentially expressed genes: *C2CD6, C7orf57, PIP5K1B*. All of them were downregulated in cases. None of them were previously found deregulated. No GO or KEGG pathways were overrepresented. As only two controls were females, the power to detect statistically significant differences after FDR correction is lower than in the global comparison. In fact, 13/19 upregulated genes in the global comparison had an unadjusted p < 0.05 and showed an upregulated profile comparing female cases with female controls (*C14orf180, CTSB, GPC6, HEPH, HSPG2, LAMP2, LMF1, LMX1B, MECOM, SELM, TNFRSF10D, VPREB3, WDR86*). In the same line, 47/58 downregulated genes in the global comparison had a non-corrected p < 0.05 (*ADAMTSL3, AIM2, AMDHD1, ANP32E, APITD1, ASPG, ASPM, ATAD5, AURKA, AURKB, BLM, BORA, BRCA1, BRCA2, BRIP1, C14orf80, C16orf59, C2CD5, C8orf48, CASC1, CASC5, CASP8, CATSPER3, CDC25C, CENPA, CENPE, CENPI, CENPI, CENPK, CEP128, CFAP46, CKAP2, CLSPN, CMPK2, CSF2RA, CYP2J2, DDIAS, DDX60, DHTKD1, DSCAM, DSCC1, DUSP19, ECT2, EFCAB11, EIF2AK2, EMP1, ERI2*).

### Male comparison

We used information from melanocyte-keratinocyte co-cultures derived from healthy skin from male melanoma cases (N=7) and male controls (N=5).

Whole transcriptome expression data analysis comparing male cases vs. male controls showed 943 differentially expressed genes, 471 upregulated and 472 downregulated genes (see following pages). Only the interferon-gamma-mediated signaling pathway GO:0060333 was overrepresented.

From the upregulated genes in this comparison, 7 were previously identified in the global comparison (*C14orf180, CCDC88C, IRX1, KLRG2, LINGO2, RNVU1-18, TMEM145*). From the downregulated genes, 22 were previously identified in the global comparison (*ACLY, ACTR3, AMDHD1, APC, APITD1, ARPC2, C14orf80, C16orf59, C6orf211, CASC1, CASP8, CATSPER3, CENPQ, CFAP46, COMMD8, CSE1L, DDX60, DNAAF3, DUSP19, ECT2, EIF2AK2, ERI2*).

From the significant genes in the overall comparison, 16/19 upregulated and 56/58 downregulated had an unadjusted p-value < 0.05 in this comparison (*TMEM145, C14orf180, CCDC88C, GPC6, HEPH, HSPG2, IRX1, KLRG2, LINGO2, LMX1B, MECOM, RNVU1-18, SELM, TNFRSF10D, VPREB3, WDR86* common up and *ACLY, ACTR3, ADAMTSL3, AIM2, AMDHD1, ANP32E, APC, APITD1, ARPC2, ASPG, ASPM, ATAD5, AURKA, AURKB, BEND6, BLM, BORA, BRCA1, BRCA2, BRIP1, C14orf80, C16orf59, C1orf112, C2CD5, C6orf211, C8orf48, CASC1, CASC5, CASP8, CATSPER3, CDC25C, CENPE, CENPK, CENPQ, CEP128, CFAP46, CKAP2, CLSPN, CMPK2, COMMD8, CSE1L, CSF2RA, CYP2J2, DDIAS, DDX60, DHTKD1, DMC1, DNAAF3, DSCAM, DSCC1, DUSP19, ECT2, EFCAB11, EIF2AK2, EMP1, ERI2* common down, respectively).

List of genes upregulated in the male comparison:

*AATK, ABCC8, ABHD16B, ACR, ACTL6B, ACTR3BP5, ADRBK1, ALPPL2, ANGPT4, ANKRD20A1, AQP8, ARMC5, ASB13, ASB18, ASCL5, ASIC4, ATXN2L, ATXN7L2, B3GALT5-AS1, BEST2, BRSK2, C10orf128, C14orf180, C16orf72, C17orf105, C18orf12, C1orf140, C1orf158, C20orf144, C20orf62, C21orf58, C22orf15, C2orf50, C3AR1, C9orf106, C9orf163, C9orf50, CA6, CABP5, CACNA1C, CACNG7, CACTIN, CALY, CAMKV, CATSPERD, CATSPERG, CCDC166, CCDC172, CCDC177, CCDC88C, CCL16, CCL19, CD300LG, CD3D, CD4, CD72, CD8A, CDH22, CDK5R2, CEACAM19, CES1, CFAP74, CHRNB3, CHST2, CLCN1, CLDN19, CLEC4M, CLHC1, CNGB1, CNPY1, COL11A2, COX6B2, CPAMD8, CPLX2, CRACR2A, CRACR2B, CREB3L1, CRY2, CRYBA2, CSF1, CT47A11, CTSLP2, CYGB, CYP26C1, CYP2F1, CYP4F62P, CYP4F8, DAB1, DCST2, DES, DNAAF2, DOT1L, DPEP3, DRP2, DUSP15, ECEL1, EML6, EMX1, EN2, EPS8L3, EPWW6493, FAM118A, FAM131C, FAM138A, FAM178B, FAM180B, FAM181A, FAM225A, FAM57B, FAM99A, FBXL18, FBXO17, FER1L6-AS2, FOXH1, FOXI3, FOXP4, FSCN2, GAST, GATSL2, GDF7, GFY, GIPR, GLI4, GML, GNG7, GPR119, GPR3, GPR97, GPSM3, GRIN3A, GSTA3, GSX1, GTF3C5, GYPC, GZMM, HAPLN2, HDGFRP2, HHLA2, HLA-DRB3, HMGA2, HMHB1, HMX2, HTATSF1P2, HTR1B, IAPP, IGF2BP2-AS1, IKZF1, IP6K1, IQGAP2, IQSEC3, IRX1, ITGA7, KANK3, KBTBD13, KCNMB1, KCNQ4, KCTD19, KGFLP2, KIF26B, KIR3DL2, KLHL29, KLRG2, KMT2B, KMT2E-AS1, KRT3, KRT85, KRTAP10-8, KRTAP19-7, KRTAP20-2, KRTAP20-3, KRTAP5-10, KRTAP5-11, KRTAP5-2, KRTAP9-1, LAMB1, LEUTX, LFNG, LINC00106, LINC00260, LINC00322, LINC00494, LINC00574, LINC00689, LINC00881, LINC00884, LINC00925, LINC00989, LINC00994, LINC01088, LINC01104, LINC01121, LINC01237, LINC01301, LINC01310, LINC01341, LINC01529, LINC-ROR, LINGO2, lnc-AC118344.1-1, lnc-BCKDHB-1, lnc-BMP7-2, lnc-BRD3-1, lnc-C16orf42-2, lnc-C9orf69-2, lnc-CBLB-4, lnc-CBWD5-2, lnc-CDH4-1, lnc-CRIPAK-1, lnc-CTTNBP2-1, lnc-DIO2-3, lnc-GDF10-2, lnc-GLIPR1-3, lnc-HES1-3, lnc-NAV1-3, lnc-PABPC4-2, lnc-QPCT-2, lnc-RTL1-2, lnc-TM4SF4-2, lnc-TMC7-1, lnc-TSHZ1-1, lnc-UXS1-4, LOC100128002, LOC100128882, LOC100129129, LOC100130152, LOC100130238, LOC100130264, LOC100130285, LOC100130540, LOC100130761, LOC100130768, LOC100130857, LOC100132874, LOC100133091, LOC100133286, LOC100289580, LOC100499194, LOC100996291, LOC101059906, LOC101927764, LOC101927910, LOC101928738, LOC101928787, LOC101930506, LOC102723701, LOC145845, LOC149950, LOC151484, LOC158435, LOC220729, LOC283335, LOC284454, LOC284933, LOC286087, LOC286382, LOC389033, LOC389273, LOC392196, LOC400863, LOC439933, LOC440028, LOC642335, LOC642947, LOC644277, LOC645427, LONRF3, LRP2, LRRC26, LTK, LY6G6D, LY9, LYZL1, LZTS2, MAFIP, MAGEA6, MEF2BNB, MEFV, MEX3D, MGC45922, MMP17, MOG, MOGAT3, MOP-1, MORN1, MOS, MPZ, MSI1, MTMR14, MUC8, MUSK, MYBPH, MYH14, MYL4, MYO3A, MYOG, NEGR1, NKG7, NKX1-2, NLRC3, NNAT, NPB, NPPA, NR2F1-AS1, NRG2, NUPL2, NYX, OPRM1, OR10A5, OR10H2, OR11A1, OR1E1, OR1L6, OR2AG2, OR6W1P, P2RX2, PACRG, PALM3, PAPL, PARD6G-AS1, PARVB, PATE3, PCDHGC4, PCDHGC5, PCIF1, PDE4A, PDE9A, PGA4, PIK3CD-AS1, PITX3, PLCXD2, POLR1C, POLR2H, POT1, PPDPF, PPIH, PPP1R16B, PPY, PRAC2, PRAME, PRKAG3, PRKCZ, PROP1, PRR25, PRR33, PSD, PTAFR, PTCH2, PTGDS, PTGER3, PTH2, PTMS, PYY2, RAI2, RAPH1, RARA, RASGRP2, RBAK-RBAKDN, RBM10, RBM48, RETNLB, RHBDL1, RIT2, RLTPR, RNA18S5, RNASE13, RNF222, RNVU1-18, ROPN1L, RORB, RP9P, RPL13AP17, RPL23P8, RRP7B, RTBDN, S100A5, SAMD11, SARDH, SBK2, SCGB2B2, SCGB3A1, SCUBE1, SEMA6B, SEPT7-AS1, SFTPA2, SHANK2-AS3, SHISA4, SHOX, SIGLEC14, SIM1, SIRPB1, SLC17A7, SLC18A3, SLC22A23, SLC22A8, SLC25A3P1, SLC35C2, SLC38A3, SLC6A17, SLC6A5, SLC6A6, SLC7A5P1, SMCR2, SMIM15, SNORA60, SNORA71A, SOWAHD, SPG7, SPINK7, SPOCK2, SPRN, SPRY4, SPSB4, SRGAP3, SSBP3-AS1, SYNGR4, SYNJ2, TAAR2, TAS2R60, TBC1D3L, TBXA2R, TCTEX1D4, TERT, TGIF1, TGM6, TLE1, TM6SF2, TMEM145, TMEM151A, TMEM200C, TMEM238, TMEM63B, TMEM82, TMEM8C, TNP2, TNRC18P1, TOR2A, TPSG1, TRHR, TSNARE1, TSPAN10, TYROBP, UBE2DNL, UBE2I, UGT2B10, UMAD1, UMOD, USHBP1, USP17L2, UTF1, VASN, VAX2, VGLL3, VWA7, WAS, WASH5P, WDR93, WDTC1, WNK4, XKR9, XLOC_l2_000864, XLOC_l2_006578, XLOC_l2_013383, XLOC_l2_013415, XLOC_l2_013837, YPEL4, YTHDF1, ZC3HAV1L, ZHX3, ZMIZ1-AS1, ZNF333, ZNF843.*

List of genes downregulated in male comparison:

*AASDHPPT, ABCB9, ABHD16A, ABHD3, ACBD3, ACLY, ACOT4, ACTL6A, ACTR3, ADI1, ADNP, ADSS, AFAP1L2, AGK, AIFM3, AKAP10, AKR1E2, ALDH1A3, AMDHD1, ANKIB1, ANKRD19P, ANKRD42, ANXA1, ANXA8L1, AP1AR, APC, APITD1, APOBR, APOL6, ARHGAP17, ARHGEF3, ARPC2, ARSB, ARSD, ASAP2, ATG10, ATPIF1, ATXN7L1, B4GALT6, BABAM1, BAZ1A, BRWD1, BST2, BTN3A2, C10orf55, C12orf29, C14orf80, C16orf59, C1QTNF1-AS1, C4orf36, C5orf15, C5orf22, C6orf211, C7orf25, CAB39, CABYR, CAP2, CAPN2, CASC1, CASC2, CASP10, CASP3, CASP8, CATSPER3, CCDC103, CCDC113, CCDC138, CCDC90B, CCT8, CDC42BPA, CDK20, CDRT1, CENPQ, CEP41, CEP97, CFAP46, CFAP58, CFDP1, CHM, CNGA1, COL22A1, COMMD4, COMMD8, COX15, CPNE8, CREM, CSE1L, CSRNP3, CSTF2, CTNNA3, CWF19L2, CXCL10, CXCL11, CYP4V2, DAAM1, DARS2, DCLRE1A, DDX23, DDX60, DEPDC5, DNAAF3, DNHD1, DPH6, DPH7, DUS4L, DUSP19, ECT2, EDAR, EFR3A, EGF, EHD4, EIF2AK2, EIF4A1, ELAC1, ELF1, EME2, EPB41L4A-AS2, ERBB2IP, ERCC8, ERI2, ERICH5, EXO5, EXOC2, EXOC6, EXTL2, FAM169A, FAM221A, FAN1, FANK1, FASTKD3, FBXO34, FERMT1, FIGNL1, FKTN, FMO4, FTCDNL1, FUCA1, G2E3, GALNT12, GBP1, GCA, GDAP2, GDPD1, GEN1, GFM2, GLCE, GMCL1, GNG10, GOLT1A, GPR160, GPRC5C, GSAP, GSTCD, GYG2, H2AFY2, HCG26, HCP5, HDGFRP3, HDX, HERC5, HERC6, HFE, HIF1AN, HIST1H2AC, HIST1H2AG, HIST2H2BF, HLA-B, HLA-F, HLA-G, HLA-J, HMGN1, HMGN5, HNRNPD, HOXA3, HOXA7, HSH2D, IDH1, IGF2BP3, IL15RA, IQCG, IQCH, IRF9, ISG15, ITPKB, JAK2, KBTBD7, KCTD8, KDM3B, KIAA0753, KIAA1107, KIAA1279, KIAA1324L, KIAA1407, KIAA1468, KIAA1841, KIF24, KIF2A, KLHL12, KLHL20, KLHL41, KLHL7, KRCC1, KREMEN2, KRR1, LAMC2, LAP3, LARP1, LEKR1, LHFPL2, LIMA1, LIN52, LINC01510, LINC01560, LINGO1, LINS, LIPG, lnc-AC114947.1.1-2, lnc-AKR1C2-4, lnc-ATG2B-2, lnc-GABPA-4, lnc-INTS9-1, lnc-MMRN1-2, lnc-PPP3CA-1, lnc-PQLC2-1, lnc-SNURF-1, LOC100131472, LOC100132057, LOC101928880, LOC102723652, LOC646268, LOC81691, LOC90768, LRRC56, LRRIQ1, LUC7L2, LY6E, LYPD1, LZTFL1, M6PR, MAGI1, MAMDC2, MAP4K4, MAPK9, MARVELD1, MCCC2, MCM8, MDH1, MDM1, ME2, METTL18, MGAT2, MKS1, MOV10, MRPL16, MRPL19, MRPL27, MRPL42, MRPS11, MSL3, MSL3P1, MTCH2, MYOM2, NAT1, NBN, NCOA5, NDUFA13, NEDD4, NEIL3, NEK8, NET1, NMI, NPAT, NUDT5, NVL, OAS1, OAS3, OAZ1, ORC4, PALB2, PANK2, PARP12, PARP9, PASK, PATL2, PCTP, PDCD6, PEBP1, PEX11A, PHKB, PI4K2B, PLA2G7, PLAU, PLIN4, PLSCR1, POC1B, POLA2, PPP3CA, PRAMEF14, PRDX5, PRKCQ-AS1, PRMT9, PRR22, PRR3, PSMB7, PSMB8, PSMD5, PSME2, PTCD2, PTPLAD2, R3HCC1L, RAB8B, RAD50, RAD51C, RAD51D, RAD54B, RAG1, RALA, RANBP1, RBM47, RCC2, RECQL5, RFC5, RGS18, RHPN1-AS1, RIC3, RLN2, RNF180, RPAP3, RPGRIP1L, RPN1, RRP1B, RTKN2, RTN4, RUNDC1, S100A11, S100A2, SAMD9L, SARNP, SCP2, SEC16B, SEC23IP, SEC62, SEMA3D, SENP2, SGK3, SH3BGRL2, SH3PXD2B, SH3TC2, SHISA5, SHOC2, SIRT5, SLC16A14, SLC25A21, SLC27A2, SLC30A9, SLC35B3, SLC37A1, SLC38A9, SLC39A10, SLFN12, SLX4, SNAP23, SNAPC3, SNRNP35, SNX10, SNX19, SNX4, SNX6, SOAT1, SPATA5, SPATS2L, SPCS2, SPIN3, SRP14, STARD3NL, STMN1, STON1-GTF2A1L, STPG1, STS, SUOX, SWT1, SYPL1, SYTL4, TAP1, TASP1, TCEAL1, TCEAL4, TCERG1, TCF20, TDRKH, TGIF2, THAP10, TIMM21, TIMMDC1, TLR3, TM7SF3, TMC5, TMEM106A, TMEM131, TMEM17, TMEM182, TMEM27, TMEM50A, TMLHE, TNIK, TNNI3, TOR3A, TPTE2P5, TRAPPC11, TRAPPC13, TRIM21, TRIM22, TRIM24, TRIM36, TRIM37, TRMT10A, TRMT6, TRRAP, TSGA10, TSNAXIP1, TSPO, TSSC1, TTC26, TTC30B, TTC9, TUG1, TWF1, TXNDC9, UBA6, UBE2L6, UEVLD, USP14, USP18, USP24, USP49, USP8, USP9Y, UTP14C, VARS2, VOPP1, VPS45, VPS53, WDHD1, WDR11, WDR36, WDR63, WDR78, WDR89, WDR92, XKR6, XLOC_l2_000297, XRRA1, ZBTB8A, ZC3H18, ZC3H7A, ZFP28, ZFP69, ZNF135, ZNF140, ZNF28, ZNF300, ZNF311, ZNF347, ZNF439, ZNF451, ZNF684, ZNF700, ZNF702P, ZNF75D, ZNF839, ZRANB2, ZWILCH.*

### Female vs. male comparison

We used information from melanocyte-keratinocyte co-cultures derived from healthy skin from females (N=11) and males (N=12) individuals without considering the case/control status.

Whole transcriptome expression data analysis comparing females vs. males independently of the disease status, showed 19 differentially expressed genes. Four genes were upregulated in females *(lnc-CHIC1-2, LOC441528, TSIX, XIST*) and 15 were downregulated (*DDX3Y, EIF1AY, FAM224A, GYG2, GYG2P1, KDM5D, NLGN4Y, RPS4Y1, RPS4Y2, TTTY14, TTTY15, TXLNGY, USP9Y, UTY, ZFY*). The demethylase activity GO:0032451 was overrepresented in these genes.

None of the differentially expressed genes identified in the global comparison were statistically significant in this comparison, even when looking at the unadjusted p-value.
